# Inhibiting HIF-1 signaling alleviates HTRA1-induced RPE senescence in retinal degeneration

**DOI:** 10.1186/s12964-023-01138-9

**Published:** 2023-06-14

**Authors:** Wenchang Xu, Xinqi Liu, Wenjuan Han, Keling Wu, Minglei Zhao, Tingfang Mei, Bizhi Shang, Jinwen Wu, Jingyi Luo, Yuhua Lai, Boyu Yang, Yehong Zhuo, Lin Lu, Yizhi Liu, Xiao-li Tian, Ling Zhao

**Affiliations:** 1 State Key Laboratory of Ophthalmology, Zhongshan Ophthalmic Center, Guangdong Provincial Key Laboratory of Ophthalmology and Visual Science, Sun Yat-sen University, Guangzhou, 510060 China; 2 Guangdong Province Key Laboratory of Brain Function and Disease, Zhongshan School of Medicine, Sun Yat-sen University, Guangzhou, China; 3 Guangzhou Regenerative Medicine and Health Guangdong Laboratory, Guangzhou, China; 4 Research Unit of Ocular Development and Regeneration, Chinese Academy of Medical Sciences, Guangzhou, China; 5 Aging and Vascular Diseases, Human Aging Research Institute (HARI), School of Life Science, Jiangxi Key Laboratory of Human Aging, Nanchang University, Nanchang, China

**Keywords:** HTRA1, Hypoxia, HIF1α, Retinal pigment epithelium, Cell senescence, Age-related macular degeneration

## Abstract

**Background:**

Age-related macular degeneration (AMD), characterized by the degeneration of retinal pigment epithelium (RPE) and photoreceptors, is the leading cause of irreversible vision impairment among the elderly. RPE senescence is an important contributor to AMD and has become a potential target for AMD therapy. HTRA1 is one of the most significant susceptibility genes in AMD, however, the correlation between HTRA1 and RPE senescence hasn’t been investigated in the pathogenesis of AMD.

**Methods:**

Western blotting and immunohistochemistry were used to detect HTRA1 expression in WT and transgenic mice overexpressing human HTRA1 (hHTRA1-Tg mice). RT-qPCR was used to detect the SASP in hHTRA1-Tg mice and ARPE-19 cells infected with HTRA1. TEM, SA-β-gal was used to detect the mitochondria and senescence in RPE. Retinal degeneration of mice was investigated by fundus photography, FFA, SD-OCT and ERG. The RNA-Seq dataset of ARPE-19 cells treated with adv-HTRA1 versus adv-NC were analyzed. Mitochondrial respiration and glycolytic capacity in ARPE-19 cells were measured using OCR and ECAR. Hypoxia of ARPE-19 cells was detected using EF5 Hypoxia Detection Kit. KC7F2 was used to reduce the HIF1α expression both in vitro and in vivo.

**Results:**

In our study, we found that RPE senescence was facilitated in hHTRA1-Tg mice. And hHTRA1-Tg mice became more susceptible to NaIO_3_ in the development of oxidative stress-induced retinal degeneration. Similarly, overexpression of HTRA1 in ARPE-19 cells accelerated cellular senescence. Our RNA-seq revealed an overlap between HTRA1-induced differentially expressed genes associated with aging and those involved in mitochondrial function and hypoxia response in ARPE-19 cells. HTRA1 overexpression in ARPE-19 cells impaired mitochondrial function and augmented glycolytic capacity. Importantly, upregulation of HTRA1 remarkably activated HIF-1 signaling, shown as promoting HIF1α expression which mainly located in the nucleus. HIF1α translation inhibitor KC7F2 significantly prevented HTRA1-induced cellular senescence in ARPE-19 cells, as well as improved the visual function in hHTRA1-Tg mice treated with NaIO_3_.

**Conclusions:**

Our study showed elevated HTRA1 contributes to the pathogenesis of AMD by promoting cellular senescence in RPE through damaging mitochondrial function and activating HIF-1 signaling. It also pointed out that inhibition of HIF-1 signaling might serve as a potential therapeutic strategy for AMD.

Video Abstract

**Supplementary Information:**

The online version contains supplementary material available at 10.1186/s12964-023-01138-9.

## Background

Age-related macular degeneration (AMD), the leading cause of irreversible vision impairment in the elderly, is characterized by the degeneration of retinal pigment epithelium (RPE) and photoreceptors [1, 2]. Age, environmental factors and genetic predisposition can influence the risk of developing AMD [1]. Genome-wide association study (GWAS) has identified more than 34 susceptibility loci for AMD and variants in *ARMS2/HTRA1* are a major genetic risk of AMD [3]. Thus, studying the underlying mechanisms of the main susceptibility genes in AMD will contribute to finding out potential therapies.

*HtrA1*, locus at 10q26 with *ARMS2* in high linkage disequilibrium, encoding a secretory protein belonging to the serine protease family [4, 5]. HtrA1 has been reported to be elevated in RPE cells, sub-RPE deposits (drusen), CNV lesions and aqueous humor of AMD patients [6–8]. Also, overexpressing human HTRA1 in the RPE of mice induces AMD-like phenotype [9]. In addition, FHTR2163, an anti-HtrA1 antibody, has been developed as a potential treatment for dry AMD in a Phase I study [10, 11]. Growing evidence shows that elevated HTRA1 in RPE plays a vital role in the pathogenesis of AMD, but the underlying mechanism is still largely undefined.

RPE senescence plays a central role in the etiology of AMD [12, 13]. Transgenic mice with HTRA1 overexpressing exhibited AMD-like phenotype when they were old [14]. These findings suggested that the upregulation of HTRA1 expression might be closely related to cell aging or senescence. However, the relationship between HTRA1 and RPE senescence in retinal degeneration hasn’t been investigated.

Here, we elucidated that HTRA1 contributes to AMD by promoting RPE senescence through damaging mitochondrial function and activating HIF-1 signaling. These findings will enhance a better understanding of AMD pathogenesis, as well as provide potential therapeutic strategy for AMD.

## Methods

### Mice

All the animal procedures were approved by the Animal Ethical Committee at Zhongshan Ophthalmic Center, Sun Yat-sen University (Guangzhou, China), and all the uses of animals were performed in accordance with the Association for Research in Vision and Ophthalmology (ARVO) statement. C57BL/6J wild-type (WT) mice and transgenic mice with human *HTRA1* knock-in (hHTRA1-Tg mice) were obtained from GemPharmatech Co., Ltd. (Jiangsu, China). To generate hHTRA1-Tg mice, the CAG-h*HTRA1* gene fragment was inserted into the H11 locus of C57BL/6J mice. gRNA and a homologous recombination vector were designed and constructed. Cas9, gRNA, and donor vector were injected into the fertilized eggs of C57BL/6J mice at the same time. Primers used for genotyping are shown in Supplementary Table 1.

### NaIO_3_ injection

C57BL/6J and hHTRA1-Tg mice aged 6 to 10 weeks were intraperitoneally injected with 20 mg/kg NaIO_3_, and age-matched mice injected with a similar volume of physiological saline served as controls. Eyes were enucleated at 10 days after the injection of 20 mg/kg NaIO_3_.

### KC7F2 injection

hHTRA1-Tg mice aged 6 to 10 weeks were intraperitoneally injected with 10 mg/kg KC7F2 for 3 days before intraperitoneally injected with 20 mg/kg NaIO_3_. After treated with 20 mg/kg NaIO_3_, mice were intraperitoneally injected with 10 mg/kg KC7F2 for 3 days.

### Anesthesia for electroretinogram (ERG), fundus photography, fundus fluorescein angiography (FFA) and spectral-domain optical coherence tomography (SD-OCT)

For all in-vivo experiments, mice were anesthetized by intraperitoneal injection of 1% pentobarbital sodium at 70 mg/kg body weight. Each animal was placed on a heating pad to recover.

### Electroretinogram (ERG)

Retinal function was assessed using the Celeris system (Diagnosys, Inc.) paired with the Espion software (Diagnosys, Inc.). Flash ERGs were performed at 4 days after 20 mg/kg NaIO_3_ injection and the procedures were as previously described [15]. Animals were dark-adapted overnight, anesthetized under red light (660 nm), and placed on a heated (37 °C) platform to maintain body temperature. Eyes were dilated with 1% tropicamide, locally anesthetized with 0.5% proparacaine hydrochloride (Alcaine), and hydrated with physiological saline. Ag/AgCl corneal stimulators were placed on either eye. Animals were subjected to a flash stimulus via the corneal stimulators. A total of 100 traces were recorded for each eye. The flash stimulus was presented at a frequency of 1 Hz and an intensity of 0.05 cd·s/m^2^. Data acquisition was conducted at a frequency of 2000 Hz for a sweep duration of 10 ms pre stimulus to 300ms post stimulus. For the acquisition of c-waves, the eyes were flashed with light intensities of 150 cd·s/m^2^ for 100 ms.

### Fundus photography, FFA and SD-OCT

For all *in-vivo* imaging, mice were anesthetized as described above. Pupils have dilated with 1% tropicamide. Fundus examinations and FFA have been performed with MICRON IV small animal retinal imaging microscope (Phoenix Research Laboratories). Mice were injected intraperitoneally with 2% fluorescein sodium 0.12 mL/20 g immediately before imaging. FFA images were captured from 1 to 10 min every 30 s. SD-OCT was taken with an HRA-OCT device, Spectralis from Heidelberg Engineering (Heidelberg). FFA and OCT images were captured with a 30° angle of view.

### Immunofluorescence

For frozen sections, enucleated eyeballs were fixed in 4% paraformaldehyde at 4 °C overnight and transferred to 30% sucrose at 4 °C overnight, then embedded in trays filled with OCT and stored at − 80 °C until sectioning. Eyeballs were sectioned at 10 μm along the sagittal plane. Cryosections were permeabilized in 0.5% Triton X-100/PBS for 10 min, blocked in 5% BSA in PBS for 1 h at room temperature, then incubated with rabbit polyclonal anti-HTRA1 antibody (1:100, Proteintech, 55011-1-AP) overnight at 4 °C. After rinsing, the sections were incubated with secondary antibodies donkey anti-Rabbit IgG Alexa Fluor 555 (1:500, Invitrogen, A31572) for 2 h at room temperature and counterstained with DAPI (1:3000, Sigma, D8417). Images were captured by a laser scanning confocal microscope (Carl Zeiss, LSM980).

### Transmission electron microscopy (TEM)

Enucleated eyes were immediately fixed in 2.5% glutaraldehyde in 0.1 M sodium cacodylate buffer (pH 7.4) (Servicebio, G1102-100ML) and dissected into small pieces. TEM was performed as previously described [16].

### Hematoxylin and eosin staining

For histology, mouse eyes were immersion-fixed overnight in FAS fixative (Wuhan Servicebio technology CO., LTD, China) at room temperature and then dehydrated in an ethanol series. Samples were embedded in paraffin and sectioned at 5 μm for hematoxylin and eosin (H&E) staining and immunofluorescence. Paraffin sections were deparaffinized by dipping 5 min in xylene followed by rehydration in dipping 2 min in each of 100%, 95%, and 80% ethanol. Slides were incubated in hematoxylin solution for 1 min, rinsed in water for 10 min, and then incubated in eosin solution for 10 s. Slides were rinsed in water and dehydrated by dipping 2 min in 80%, 95%, and 100% ethanol and 1 min in xylene for 2 times. Coverslips were placed on slides using neutral balsam. Slides were analyzed under a light microscope (Tissue FAXS Q+, Tissue Gnostics, Austria).

### Cell culture and adenovirus infection

ARPE-19 cells (GDC0323) were purchased from the China center for type culture collection (CCTCC). Mycoplasma contamination test was performed before experiments to ensure the cells were negative. ARPE-19 cells were cultured as previously described [17]. For adenovirus infection, cells were infected with adenovirus carrying human HTRA1 gene with C terminal fused Flag/His tag (adv-HTRA1) at a multiplicity of infection (m.o.i.) of 20, null-control adenovirus (adv-NC) was used as a negative control. The incubation time varies from 24 to 48 h according to the needs of the experiments.

### Isolation and culture of primary mouse RPE cells

The method of isolation and culture of primary mouse RPE cells was as previously described [18].

### Western blot

Western blot was performed as previously described [19]. The following antibodies were used for western blotting: rabbit polyclonal anti-6×His tag antibody (1:1000, Abcam, ab9108), rabbit monoclonal RPE65 antibody (1:1000, Abcam, ab231782), rabbit polyclonal anti-HTRA1 antibody (1:1000, Proteintech, 55011-1-AP), rabbit monoclonal anti-HIF1α antibody (1:1000, CST, #36,169), rabbit polyclonal anti-GAPDH antibody (1:1000, Abclonal, AC001), mouse polyclonal β-actin antibody (1:1000, Abclonal, AC004), Histone-H3 antibody (1:1000, Proteintech, 17168-1-AP) and secondary antibodies: horseradish peroxidase linked anti-rabbit/mouse IgG (1:5000, CST, #7074/7076). Blots were visualized using secondary antibodies conjugated with horseradish peroxidase by Chemiluminescence Imaging System (UVITEC).

### RNA-seq analysis

RNA sequencing and analysis were performed by Berry Genomics Corporation. RNA was extracted from ARPE-19 cells treated with adv-NC or adv-HTRA1 for 24 h. Triplicate samples in each group were prepared and sequenced as previously described [19]. Raw data were processed and the clean data were aligned to the human reference genome using TopHat v2.0.12. The reads numbers mapped to each gene were counted using HTSeq v0.6.1. DESeq R package (1.18.0) was used to analyze differentially expressed genes and genes with an adjusted *p*-value < 0.05 found by DESeq were assigned as differentially expressed. Gene Ontology (GO) enrichment analysis of differentially expressed genes was implemented by the GOseq R package and GO terms with corrected *p*-value less than 0.05 were considered significantly enriched by differential expressed genes. The statistical enrichment of differential expression genes in KEGG pathways was tested using KOBAS software.

### Bioenergetics analysis with seahorse

Seahorse Bioscience XFe24 analyzer (Agilent) was used for the measurement of oxygen consumption rate (OCR) and extracellular acidification rate (ECAR). ARPE-19 cells (20,000 cells/well) were seeded in XF24 culture microplates for 18 h and then treated with 0µM or 10µM KC7F2 for 72 h, the last 24 h were treated with adv-NC or adv-HTRA1 together. OCR was measured with Cell Mito Stress Test kit under basal conditions followed by the sequential addition of 2.5 µM oligomycin, 2 µM FCCP and 0.9 µM rotenone & antimycin A. ECAR was measured with the Glycolysis Stress Test kit under basal conditions followed by the sequential addition of 10 mM glucose, 2 µM oligomycin, and 50 mM 2-DG. The OCR and ECAR were calculated by the Seahorse XFe24 Wave software.

### Hypoxia detection assay

EF5 Hypoxia Detection Kit, Cyanine 3 (Sigma-Aldrich, #EF5-30C3) was used to detect ARPE-19 cells’ hypoxia. After treated with adv-NC/adv-HTRA1 or 1% O_2_ for 24 h, 150 µl of 10mM EF5 was added to each well and placed into the incubator for 15 to 30 min. Cells were fixed with 4%PFA for 15 min at room temperature and washed 3 times with ttPBS. 200 µl blocking solution was added to each well and blocked at 37 ° C for 1 h. After washing 3 times with ttPBS, cells were incubated with 150 µl 75 µg/mL EF5 antibody (ELK3-51 Cyanine 3 conjugate) for 4–6 h at 4 ° C and protected from light. DAPI (1:3000, Invitrogen) was used for nuclear counterstaining. Images were captured by a laser scanning confocal microscope (Carl Zeiss, LSM980).

### Immunofluorescence microscopy

Cells seeded on glass coverslips (Thermo Fisher) were fixed with 4% paraformaldehyde for 30 min at room temperature, washed with PBS three times, permeabilized with 0.25% Triton-100 for 10 min, blocked in 5% BSA for one hour at 37 °C and then incubated with primary antibodies diluted in 3% BSA at 4 °C overnight. After being rinsed with PBS three times, the cells were incubated with secondary antibodies diluted in 3% BSA for one hour at room temperature. The following antibodies were used for immunofluorescence: rabbit monoclonal anti-HIF1α antibody (1:300, CST, #36,169), rabbit phospho-histone H2A.X (Ser139) antibody (1:300, CST, #2577) and the secondary antibody goat anti-rabbit Alexa555 IgG (1:500, Invitrogen), DAPI (Invitrogen) was used for nuclear counterstaining. Images were captured by a laser scanning confocal microscope (Carl Zeiss, LSM980).

### Intracellular reactive oxygen species (ROS) generation measurement

ARPE-19 cells were treated with adv-NC or adv-HTRA1 for 24–48 h, DCFH-DA detection kit (Beyotime) was used to determine ROS generation. Experiments were done according to the manufacturer’s instruction, the DCFH-DA fluorescence data were measured using flow cytometry.

### Quantitative real-time RT-PCR (RT-qPCR)

TRIzol Reagent (Thermo Fisher) was used to extract the total RNA and ReverTra Ace qPCR RT kit (TOYOBO, FSQ-301) was applied to the reverse transcription. RT-qPCR was performed with the LightCycler® 96 Real-Time PCR System (Roche) and the primers used are shown in Supplementary Table 2.

### Cytoplasmic and nuclear protein extraction

Nuclear and Cytoplasmic Protein Extraction Kit (Beyotime, P0027) was used for cytoplasmic and nuclear protein extraction. ARPE-19 cells were seeded 6-well plates and treated with adv-NC or adv-HTRA1 for 24 h. Experiments were done according to the manufacturer’s instructions.

### Senescence-associated-β-galactosidase (SA-β-gal)

For ARPE-19 cells treated without KC7F2, ARPE-19 cells were seeded 24-well plates and treated with adv-NC or adv-HTRA1 for 24–48 h. For ARPE-19 cells treated with KC7F2, ARPE-19 cells were seeded 12-well plates and treated with KC7F2 0µM or 10µM for 72 h and the last 24 h were treated with adv-HTRA1 or adv-NC together. A senescence β-Galactosidase staining kit (CST) was used to detect β-galactosidase activity. Experiments were done according to the manufacturer’s instructions. Check the cells under a microscope for the development of blue color and 5–10 fields were selected for positive staining cell counting for each group. For frozen sections, experiments were done according to the manufacturer’s instructions. Images were captured by Tissue FAXS (TissueGnostics, Austria).

### Statistical analysis

Data were expressed as mean ± SEM. Student’s t-test and one-way ANOVA were used to evaluate differences between groups. The Chi-square test was used to evaluate the proportion of retinal degeneration in hHTRA1-Tg and WT mice induced by 20 mg/kg NaIO_3_. A value of *P* < 0.05 was considered to be statistically significant. ns, no significance; *, *P* < 0.05; **, *P* < 0.01; ***, *P* < 0.001; ****, *P* < 0.0001. Statistical analysis was performed using GraphPad Prism 6 (GraphPad Software). ImageJ software was used to quantify the expression of the protein.

## Results

### HTRA1 overexpression accelerated the senescence of retinal pigment epithelium in mice

**Fig. 1:**
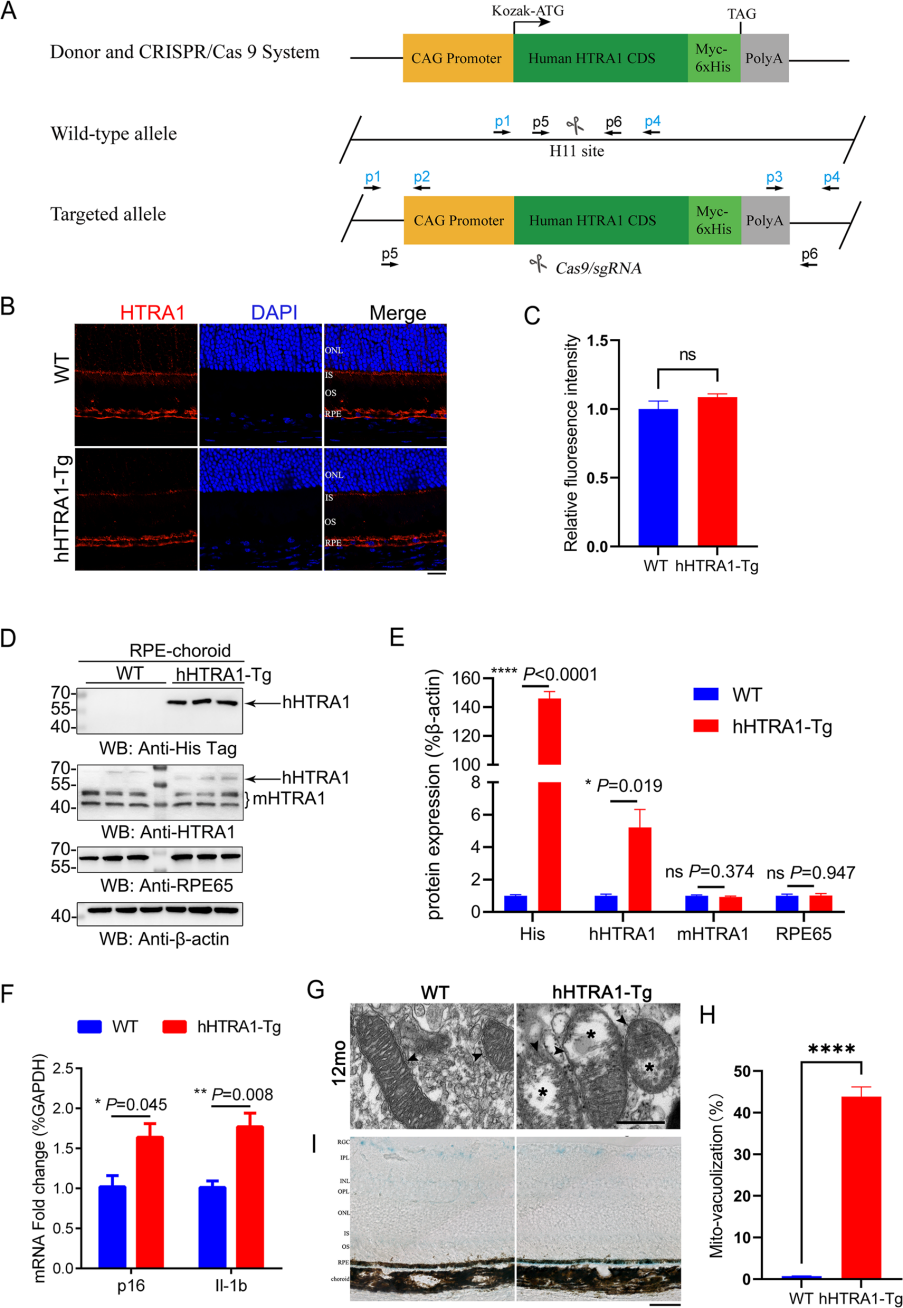
HTRA1 overexpression aggravated RPE senescence in mice. **A** The strategy for creating transgenic mice with human *HTRA1* knock-in (hHTRA1-Tg). **B** Immunofluorescent analysis of retinal HTRA1 expression in 6- to 8-week-old WT and hHTRA1-Tg mice (the scale bar is 20 μm) (*n* = 3). ONL, outer nuclear layer; IS, photoreceptor inner segment; OS, photoreceptor outer segment; RPE, retinal pigment epithelium. **C** Statistical analysis of relative fluorescence intensity in B. **D** Western blot analysis of HTRA1 and RPE65 expression in RPE-choroid of 6- to 8-week-old WT and hHTRA1-Tg mice. **E** Statistical analysis of the relative expression of HTRA1 and RPE65 in each group of D. **F** RT-qPCR analysis of p16 and Il-1β expression in RPE-choroid of 6- to 8-week-old WT and hHTRA1-Tg mice. **G** TEM images of mitochondria in RPE (the scale bar is 500 nm, *n* = 3). Arrowheads indicated normal mitochondria and asterisks indicated vacuolated changes in mitochondria. **H** Statistical analysis of mitochondrial vacuolization in RPE. **I** Retinal SA-β-gal staining in 12-month-old WT and hHTRA1-Tg mice (the scale bar is 50 μm, *n* = 3). RGC, retinal ganglion cell; IPL, inner plexiform layer; INL, inner nuclear layer; OPL, outer plexiform layer; ONL, outer nuclear layer; IS, photoreceptor inner segment; OS, photoreceptor outer segment; RPE, retinal pigment epithelium

To explore whether elevated HTRA1 affects RPE senescence, we generated a transgenic mouse with human *HTRA1* knock-in (hHTRA1-Tg) (Fig. 1A, S1). The immunofluorescence results showed HTRA1 was expressed in the RPE of WT and hHTRA1-Tg mice (Fig. 1B-C). Compared with age-matched wild-type mice, human HTRA1 with C-terminal his tag was found overexpressed in retina and RPE-choroid of hHTRA1-Tg mice, as detected by the anti-6×his tag antibody and anti-HTRA1 antibody (Fig. 1D-E, S2). We tested the mRNA expression of p16 and Il-1b in RPE-choroid of 6 to 8-week-old WT and hHTRA1-Tg mice and found the expression were increased (Fig. 1F). And in 12-month-old mice, normal mitochondria (Fig. 1G, arrowheads) were found in the RPE of WT mice, while vacuolated changes in mitochondria (Fig. 1G, asterisks, Fig. 1H) were detected in the RPE of hHTRA1-Tg mice, as well as enhanced senescence-associated-β-galactosidase (SA-β-gal), a known characteristic of senescent cells (Fig. 1I). These results showed that the overexpression of HTRA1 could induce RPE senescence in mice.

### The hHTRA1-Tg mice became more susceptible to NaIO_3_in the development of retinal degeneration

**Fig. 2:**
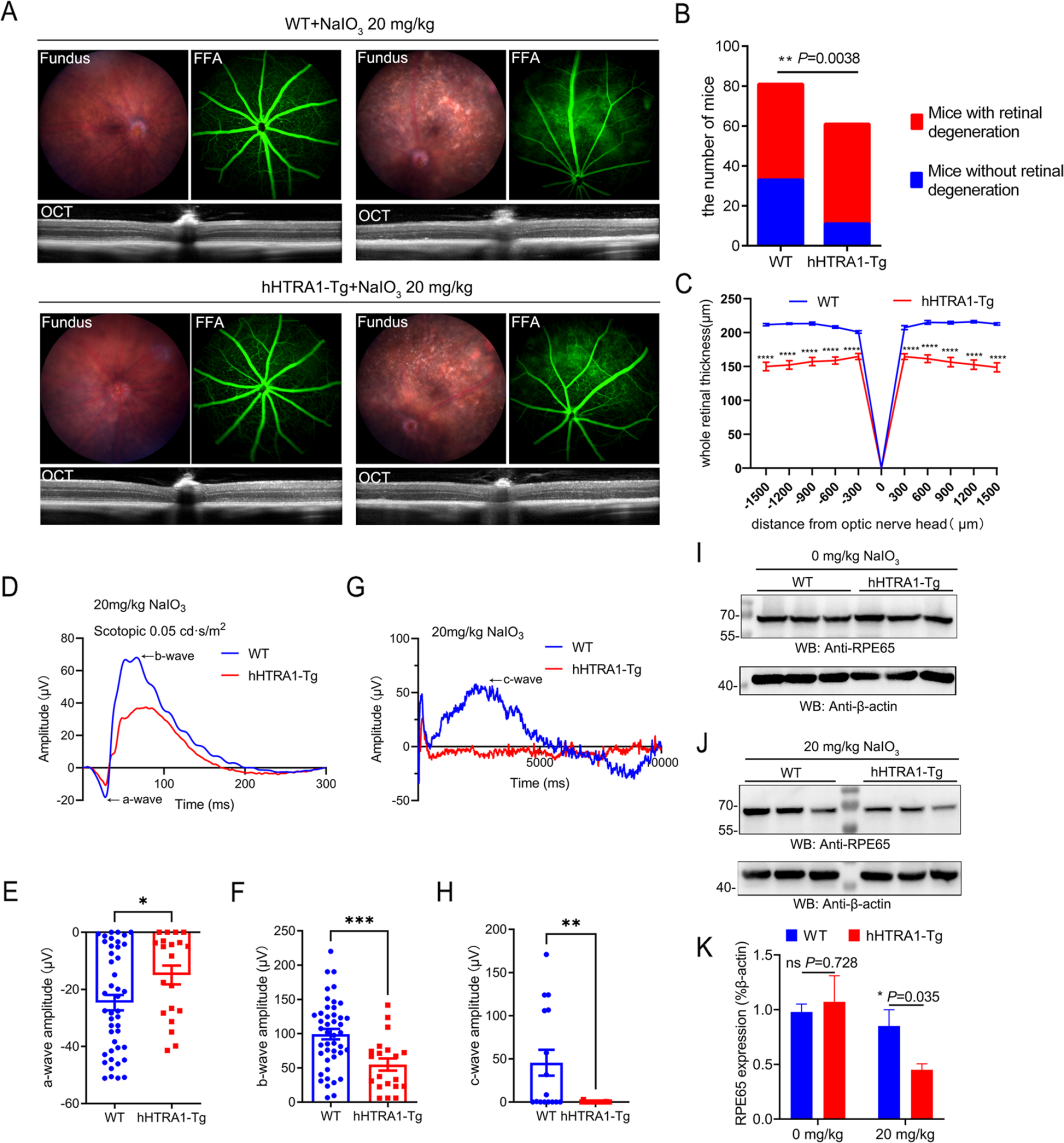
hHTRA1-Tg mice became more susceptible to NaIO_3_ in the development of retinal degeneration. **A** Representative fundus image, FFA and OCT of WT and hHTRA1-Tg mice with an intraperitoneal injection of 20 mg/kg NaIO_3_ or similar volume of physiological saline. The left set showed the mice without obvious retinal degeneration and the right showed the mice with retinal degeneration. **B** The histogram showed the number of WT and hHTRA1-Tg mice with or without retinal degeneration after being treated with 20 mg/kg NaIO_3_ (82 WT mice was detected, 27 out of 31 female mice and 21 out of 51 male mice showed retinal degeneration. 61 Tg mice was detected, 25 out of 30 female mice and 25 out of 31 male mice showed retinal degeneration). **C** Statistical analysis of the whole retinal thickness. **D** Representative a-wave and b-wave ERG traces of 6- to 10-week-old hHTRA1-Tg (*n* = 10) and WT mice (*n* = 22) on the fourth day after intraperitoneal injection of 20 mg/kg NaIO_3_. Flash intensity was 0.05 cd·s/m^2^. **E** Statistical analysis of a-wave amplitudes for WT and hHTRA1-Tg mice (*P* = 0.038). **F** Statistical analysis of b-wave amplitudes for WT and hHTRA1-Tg mice (*P* = 0.001). **G** Representative c-wave ERG traces of 6- to 10-week-old hHTRA1-Tg (*n* = 9) and WT mice (*n* = 8) on the fourth day after intraperitoneal injection of 20 mg/kg NaIO_3_. **H** Statistical analysis of c-wave amplitudes for WT and hHTRA1-Tg mice (*P* = 0.003). **I** Western blot analysis of RPE65 expression in RPE-choroid of WT and hHTRA1-Tg mice. **J** Western blot analysis of RPE65 expression in RPE-choroid of WT and hHTRA1-Tg mice treated with 20 mg/kg NaIO_3_. **K**. Statistical analysis of the relative expression of RPE65 in each group of I, J

There was little obvious retinal degeneration observed by ERG, fundus photography, FFA and SD-OCT in both wild-type and hHTRA1-Tg mice at 6–10 weeks (Fig. S3). Hematoxylin and eosin staining (H&E staining) of 8-week and 12-month-old hHTRA1-Tg mice didn’t show any structure changes compared with age-matched WT mice (Fig. S4). Sodium iodate (NaIO_3_, SI) is an oxidative toxic agent that has been widely used to build a reproducible model of dry AMD [20, 21]. We firstly treated the mice with 10 mg/kg NaIO_3_, a low dose that has been reported not cause any morphological or functional changes in mouse retina. Compared with control mice intraperitoneal injected with physiological saline, little obvious changes were found in the fundus images, FFA and OCT results of WT and hHTRA1-Tg mice in 1 month (Fig. S5, A). We then treated the mice with 35 mg/kg NaIO_3_, a high dose that has been reported causing severe retinal degeneration in a week. We observed severe retinal abnormalities in all WT and hHTRA1-Tg mice after intraperitoneal injection of 35 mg/kg NaIO_3_ on day 7, including significant pigmentary changes of the RPE layer found in fundus image and enhanced background fluorescence in FFA. OCT showed RPE atrophy associated with subretinal deposition and retinal thinning mainly caused by loss of outer nuclear layer (ONL) and photoreceptors, while control mice showed no effect on the retina (Fig. S5, B). We tested the expression of HTRA1 in retina and RPE-choroid of WT mice treated with 10 mg/kg or 35 mg/kg SI and found that HTRA1 in retina did not response to the stress (Fig. S6 A-B), but RPE-choroid did (Fig. S6 C-D). HTRA1 was upregulated in the 10 mg/kg SI treatment group and decreased in the 35 mg/kg SI management group in RPE-choroid (Fig. S6 C-D). Then we treated mice with 20 mg/kg SI. 10 days after intraperitoneal injection of 20 mg/kg NaIO_3_, some WT and hHTRA1-Tg mice were detected with retinal degeneration through fundus photography, FFA and OCT while some did not (Fig. 2A). Interestingly, the proportion of retinal degeneration in hHTRA1-Tg mice induced by 20 mg/kg NaIO_3_ was higher than that of WT mice (Fig. 2B). The whole retinal thickness was reduced in hHTRA1-Tg mice compared to WT mice (Fig. 2C). And 4 days after intraperitoneal injection of 20 mg/kg NaIO_3_, the a-wave and b-wave of scotopic flash ERG were reduced in hHTRA1-Tg mice compared to WT mice (Fig. 2D-F), which indicated the impairment of photoreceptors and bipolar cells were more severe in hHTRA1-Tg mice. The c-wave of ERG was also decreased (Fig. 2G, H), which indicate the RPE damage was more severe in hHTRA1-Tg mice. To further explore the RPE degeneration induced by 20 mg/kg NaIO_3_, we detected the expression of RPE65 in RPE-choroid, a key enzyme in the vertebrate visual cycle primarily expressed in RPE. There was no significant difference between WT and hHTRA1-Tg mice without NaIO_3_ treatment (Fig. 2I, K). However, when treated with 20 mg/kg NaIO_3_, the expression of PRE65 in RPE-choroid was significantly decreased in hHTRA1-Tg mice (Fig. 2J, K). These results indicated that HTRA1 overexpression aggravated NaIO_3_-induced retinal degeneration.

### HTRA1 overexpression aggravated the cellular senescence of ARPE-19 cells

**Fig. 3:**
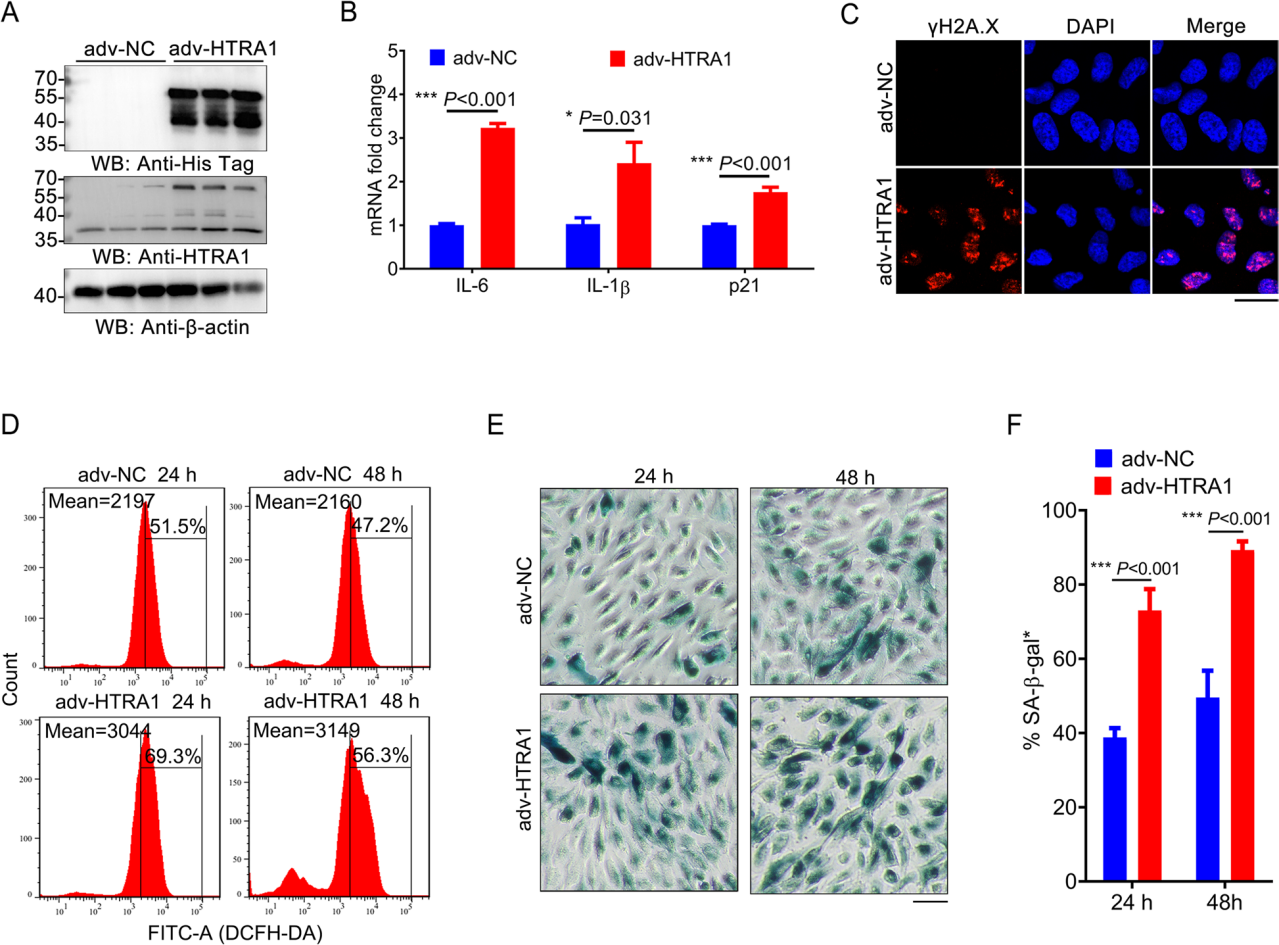
HTRA1 overexpression aggravated cellular senescence of ARPE-19 cells. **A** Western blot analysis of HTRA1 overexpression in ARPE-19 cells treated with adv-NC or adv-HTRA1 for 24 h. **B** RT-qPCR analysis of IL-1β, IL-6 and p21 expression in ARPE-19 cells treated with adv-NC or adv-HTRA1 for 24 h. **C** Immunofluorescent analysis of DNA damage by detecting the expression of γH2A.X in ARPE-19 cells treated with adv-NC or adv-HTRA1 for 24 h (the scale bar is 20 μm, *n* = 3). **D** Flow cytometry detection of ROS in ARPE-19 cells treated with adv-NC or adv-HTRA1 for 24 or 48 h. **E** SA-β-gal staining of ARPE-19 cells treated with adv-NC or adv-HTRA1 for 24 or 48 h (the scale bar is 50 μm). **F** Statistical analysis of the positive staining cells in each group in E

Then we examined the effect of elevated HTRA1 on the cellular senescence of ARPE-19 cells. We overexpressed HTRA1 with C-terminal his tag in ARPE-19 cells by adv-HTRA1. Detected by the anti-6×his tag antibody and HTRA1 antibody, HTRA1 was found overexpressed in ARPE-19 cells after being treated with adv-HTRA1 for 24 h (Fig. 3A). Upregulation of IL-6, IL-1β and p21, which involved in senescence-associated secretory phenotype (SASP), were found in ARPE-19 cells overexpressing HTRA1 (Fig. 3B). And a substantial increase in γH2A.X foci formation (biomarker for DNA double-strand break) in the nucleus was observed in ARPE-19 cells treated with adv-HTRA1 (Fig. 3C). We isolated and cultured the primary mouse RPE cells from WT mice and treated with adv-HTRA1 for 48 h. We found that γH2A.X foci were also increased in primary mouse RPE cells (Fig. S7). In addition, intracellular reactive oxygen species (ROS) level increased after 24 or 48 h treatment of adv-HTRA1 (Fig. 3D). We’ve also detected the mitochondrial ROS production using MitoSOX but it did not increase significantly (Fig. S8). Finally, we detected the accumulation of SA-β-gal in ARPE-19 cells treated with adv-NC or adv-HTRA1. Compared with the control group treated with adv-NC, the percentage of positive staining cells in the adv-HTRA1 treated group was significantly increased after 24 and 48 h, respectively (Fig. 3E-F). These results above demonstrated that overexpressed HTRA1 accelerated the senescence of retinal pigment epithelium.

### Elevated HTRA1 in ARPE-19 cells profoundly affected mitochondrial function and hypoxia-related signaling pathways

**Fig. 4:**
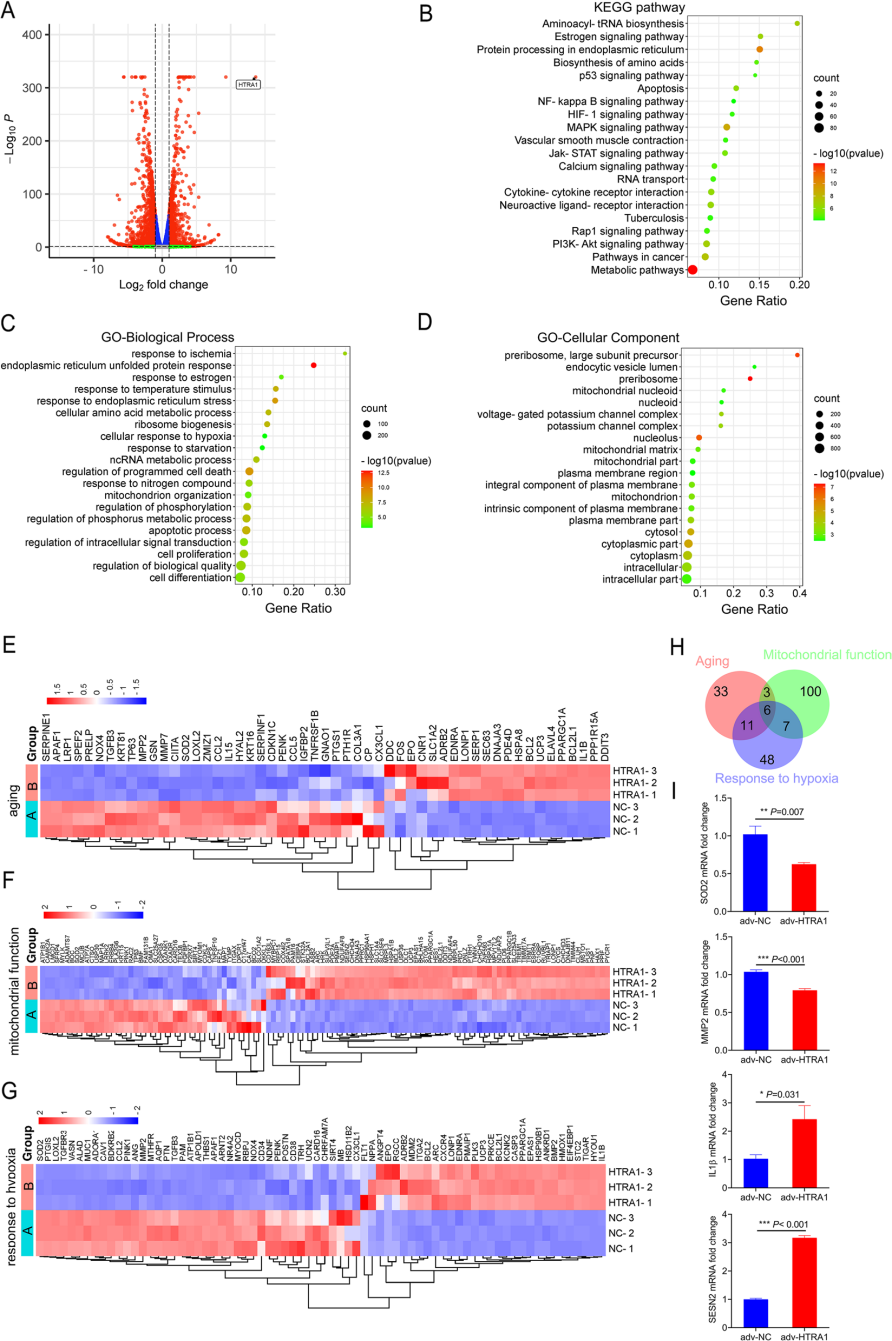
HTRA1 overexpression profoundly affected mitochondrial function and hypoxia-related signaling pathways in ARPE-19 cells. **A** Volcano plot representation of differentially expressed genes (DEGs) (log2 |fold change|> 1, log10 adjusted *p*-values < 0.05) in ARPE-19 cells treated with adv-HTRA1 versus adv-NC. **B** Bubble chart of KEGG pathway enrichment for up-regulated genes. **C**-**D** Bubble chart of GO enrichment for up-regulated genes, the distributions are summarized in two categories: biological process **C** and cellular component **D**. The color of the bubble means the significance of the corresponding pathway and the size of the bubble means the number of DEGs in this pathway. **E**-**G** Heatmap of 53 altered aging-related genes, 116 altered genes involved in mitochondrial function and 72 altered genes in response to hypoxia. **H** Venn diagram of the differentially expressed genes (DEGs) involved in aging (red), mitochondrial function (green) and response to hypoxia (blue), respectively. **I** RT-qPCR analysis of 4 differentially expressed genes (SOD2, MMP2, IL-1β and SESN2) from RNA-seq data

To explore the candidate signaling pathways involved in cellular senescence aggravated by elevated HTRA1 in RPE cells, we performed RNA-seq analysis on ARPE-19 cells infected with adv-HTRA1 or adv-NC for 24 h. Compared with control groups, 2810 differentially expressed genes (log2 |fold change|> 1, log10 adjusted p-values < 0.05) were identified in ARPE-19 cells overexpressing HTRA1. 1053 genes were significantly up-regulated and the other 1757 genes were significantly down-regulated (Fig. 4A). We performed the KEGG pathways analysis of up-regulated genes and ranked the enriched pathways according to gene ratio. We noticed that the HIF-1 signaling pathway, which is critical in the cellular response to hypoxia, was involved in the enriched pathways. Besides, the MAPK signaling pathway and PI3K-Akt signaling pathway, which play important roles in the regulation of HIF1α, were included in the KEGG pathways (Fig. 4B). We next performed GO enrichment analysis of up-regulated genes involved in biological process and cellular component (Fig. 4C-D). Cellular response to hypoxia was among the main biological process terms. Response to ischemia and response to starvation, which are closely associated with hypoxia, were also in the main biological process terms (Fig. 4C). Mitochondrial nucleoid rank fourth in the cellular component terms. Mitochondrial matrix, mitochondrial part and mitochondrion were involved in the main cellular component terms (Fig. 4D). We checked 2810 significantly differentially expressed genes annotated by GO terms and found 53 aging-related genes, 116 genes involved in mitochondrial function and 72 genes in response to hypoxia (Fig. 4E-G). There was an overlap between the three groups of altered genes (Fig. 4H). Finally, we randomly tested some differentially expressed genes (IL-1β, SOD2, MMP2 and SESN2) from RNA-seq results through RT-qPCR and confirmed that the results were verifiable (Fig. 4I). These results suggested that HTRA1 may regulate cellular senescence by affecting mitochondrial function and hypoxia signaling pathways in ARPE-19 cells.

### Elevated HTRA1 impaired mitochondrial respiration and promoted glycolysis in ARPE-19 cells

**Fig. 5:**
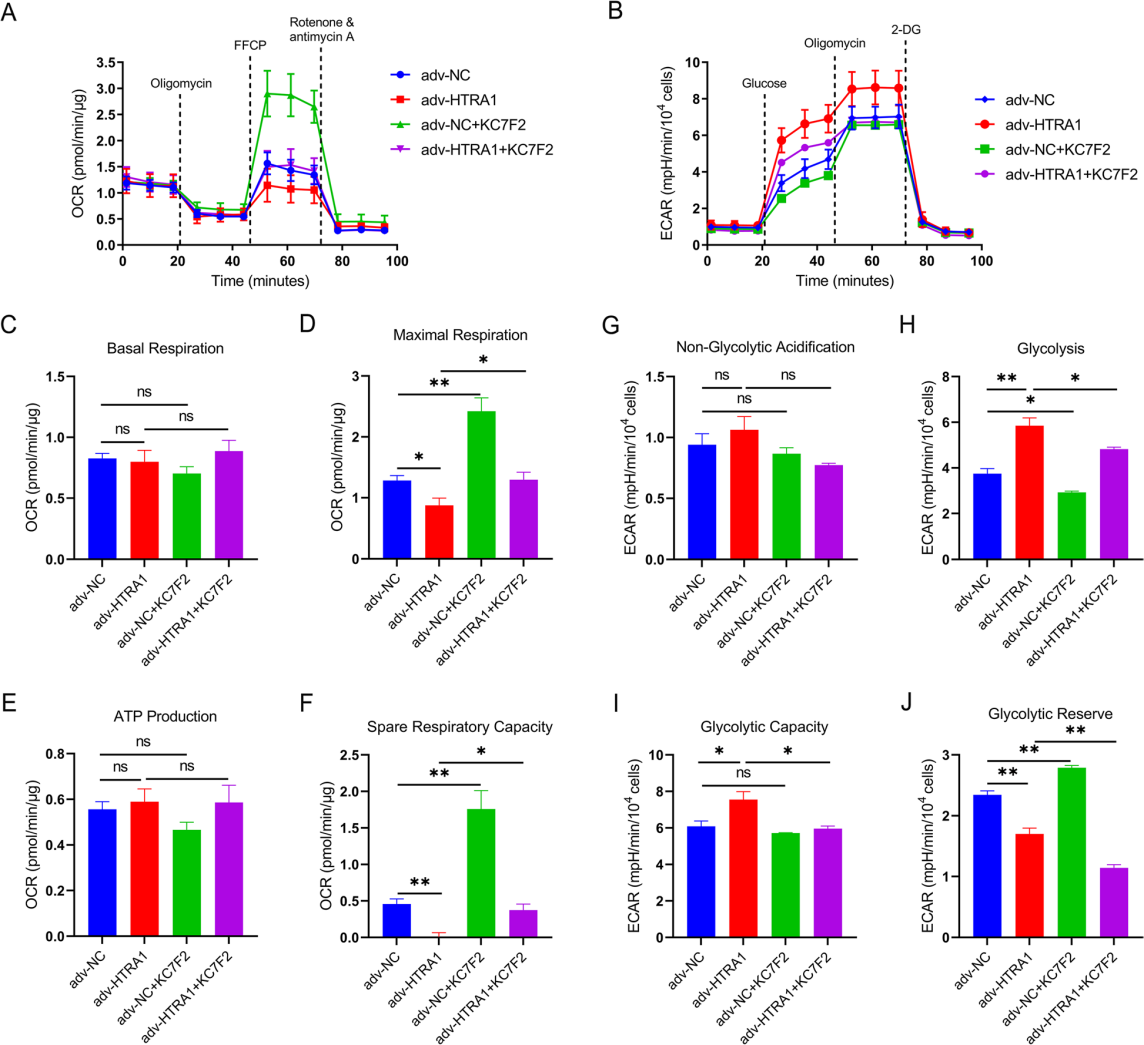
HTRA1 overexpression triggered the transition of cellular energy metabolism from oxidative phosphorylation to glycolysis in ARPE-19 cells. **A** Measurement of oxygen consumption rate (OCR) in ARPE-19 cells infected with adv-NC, adv-HTRA1, adv-NC + 10 µM KC7F2 or adv-HTRA1 + 10 µM KC7F2 respectively. Dotted lines indicated the time points of adding oligomycin, carbonyl cyanite-4 (trifluoromethoxy) phenylhydrazone (FCCP), and Rotenone/Antimycin **A**. **C**-**F** Statistical analysis of basal respiration (**C**), maximal respiration (**D**), ATP production (**E**) and spare respiratory capacity (**F**). **B** Measurement of extracellular acidification rate (ECAR) in ARPE-19 cells infected with adv-NC, adv-HTRA1, adv-NC + 10 µM KC7F2 or adv-HTRA1 + 10 µM KC7F2 respectively. Dotted lines indicated the time points of adding glucose, oligomycin and 2-DG. G-J Statistical analysis of non-glycolytic acidification (**G**), glycolysis (**H**), glycolytic capacity (**I**) and glycolytic reserve (**J**)

Mitochondrial dysfunction is a hallmark of cellular senescence which contributes to the senescence growth arrest, SASP development and resistance to cell death [22, 23]. The RNA-seq data showed that elevated HTRA1 affected mitochondrial function, we then measured the oxygen consumption rate (OCR) and extracellular acidification rate (ECAR) in APRE-19 cells. Both OCR and ECAR measurements were taken in real-time in ARPE-19 cells treated with adv-NC or adv-HTRA1 for 24 h (Fig. 5A-B). For OCR measurements, ARPE-19 cells treated with adv-HTRA1 showed significantly reduced maximal respiration (Fig. 5D) and spare respiratory capacity (Fig. 5F), but basal respiration (Fig. 5C) and ATP production (Fig. 5E) did not change. These results revealed that elevated HTRA1 impairs the mitochondrial respiration of ARPE-19 cells. For ECAR measurements, ARPE-19 cells treated with adv-HTRA1 showed significant increased glycolysis (Fig. 5H) and glycolytic capacity (Fig. 5I), but non-glycolytic acidification (Fig. 5G) did not change and glycolytic reserve (Fig. 5J) was reduced. These results indicated that elevated HTRA1 promote the process of converting glucose to pyruvate when sufficient glucose was provided and promoted the capability to use glycolysis to respond to an energetic demand when oxidative phosphorylation is impeded in ARPE-19 cells.

The RNA-seq data showed HIF-1 signaling pathway was enriched. To explore whether inhibiting HIF1α could reverse the HTRA1-induced mitochondrial respiration impairment and reduce glycolysis, we used KC7F2, a HIF1α translation inhibitor, to treat ARPE-19 cells for 72 h and measured the OCR and ECAR (Fig. 5A-B). We found that KC7F2 could improve the maximal respiration (Fig. 5D) and spare respiratory capacity (Fig. 5F), and also reduce glycolysis (Fig. 5H) and glycolytic capacity (Fig. 5I) in ARPE-19 cells treated with adv-HTRA1. Taken together, these results showed that elevated HTRA1 impaired mitochondrial respiration and promoted glycolytic in ARPE-19 cells through HIF-1 signaling. And inhibiting HIF1α could reverse the HTRA1-induced mitochondrial respiration impairment and reduce glycolysis.

### HTRA1 overexpression induced cellular hypoxia and activated the HIF-1 signaling pathway in ARPE-19 cells

**Fig. 6:**
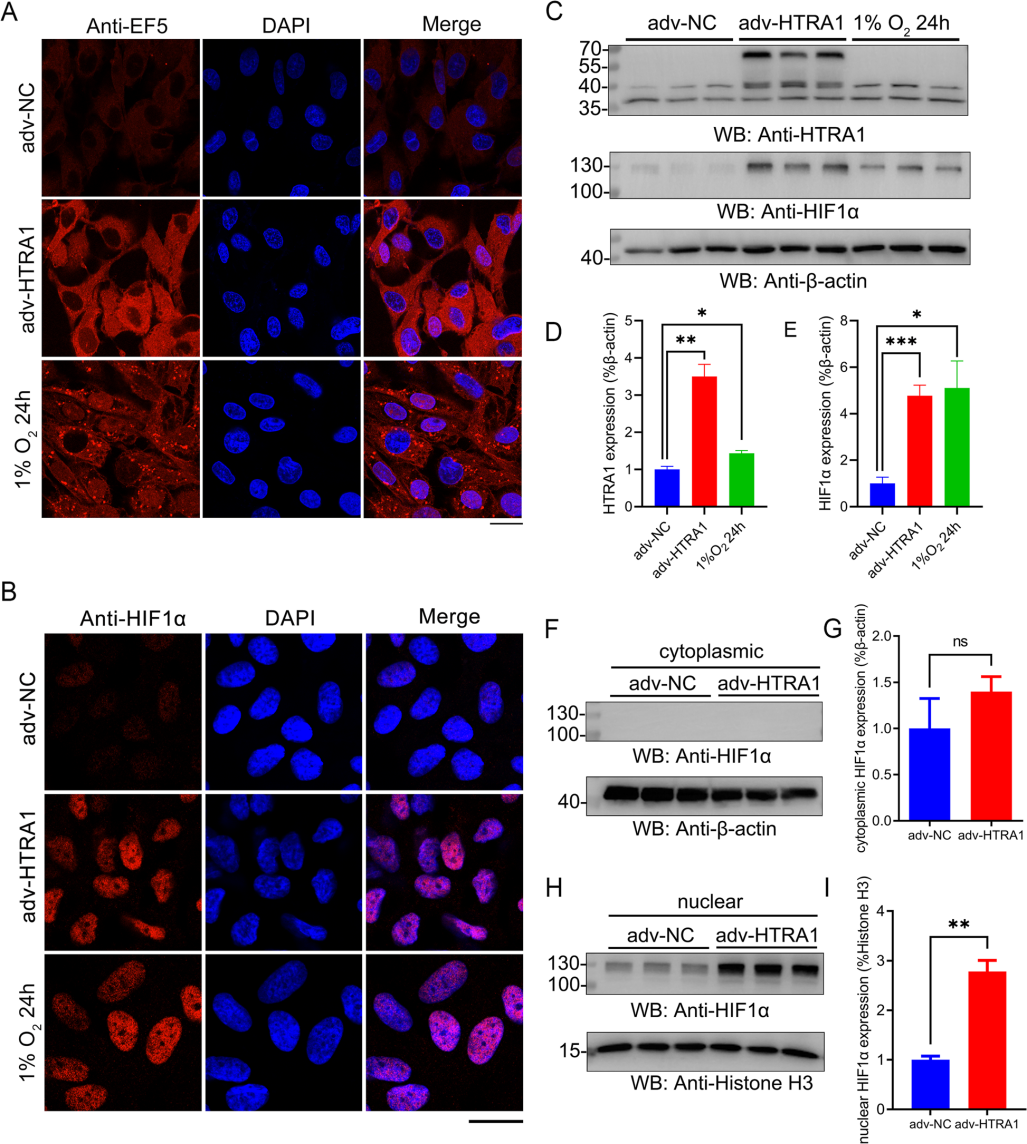
HTRA1 induced hypoxia in ARPE-19 cells. **A** Immunofluorescent analysis of EF5 expression in ARPE-19 cells treated with adv-NC or adv-HTRA1 for 24 h. Cells treated with 1% O_2_ for 24 h were used as a positive control (*n* = 3). **B** Immunofluorescent analysis of HIF1α expression in ARPE-19 cells treated with adv-NC or adv-HTRA1 for 24 h. Cells treated with 1% O_2_ for 24 h were used as a positive control (*n* = 3). **C** Western blot analysis of HTRA1 and HIF1α expression in ARPE-19 cells treated with adv-NC or adv-HTRA1 for 24 h. Cells treated with 1% O_2_ for 24 h were used as a positive control. **D** Statistical analysis of the relative expression of HTRA1. **E** Statistical analysis of the relative expression of HIF1α. **F** Western blot analysis of HIF1α expression of cytoplasmic protein extracted from ARPE-19 cells treated with adv-NC or adv-HTRA1 for 24 h. **G** Statistical analysis of the relative expression of HIF1α in cytoplasm. **H** Western blot analysis of HIF1α expression of nuclear protein extracted from ARPE-19 cells treated with adv-NC or adv-HTRA1 for 24 h. **I** Statistical analysis of the relative expression of HIF1α in nucleus

Hypoxia is a potent stressor to cause mitochondrial dysfunction with insufficient energy production and increased ROS [24]. And RNA-seq results showed that elevated HTRA1 activated hypoxia-related signaling pathways. We then detected the hypoxia in ARPE-19 cells with HTRA1 overexpression through EF5, a 2-nitroimidazole-based probe that selectively binds hypoxic cells. The immunofluorescent results showed that overexpression of HTRA1 induced hypoxia in ARPE-19 cells as 1% O_2_ did (Fig. 6A). And the HIF1α expression was upregulated in the nucleus in ARPE-19 cells with HTRA1 overexpressing (Fig. 6B). We’ve also detected the HIF1α expression in primary mouse RPE cells and found the same trend as in ARPE-19 cells (Fig. S9). We further compared the expression of HIF1α by western blot, and found HIF1α at nearly 130 kDa was significantly increased when HTRA1 was overexpressed (Fig. 6C-E). And after extracting the nuclear protein for ARPE-19 cells, we found that HIF1α mainly expressed in the nucleus (Fig. 6F-I). These findings showed that HTRA1 overexpression induced cellular hypoxia and activated the HIF-1 signaling pathway in ARPE-19 cells.

### Cellular senescence induced by HTRA1 overexpression could be halted by the HIF-1α translation inhibitor KC7F2

**Fig. 7:**
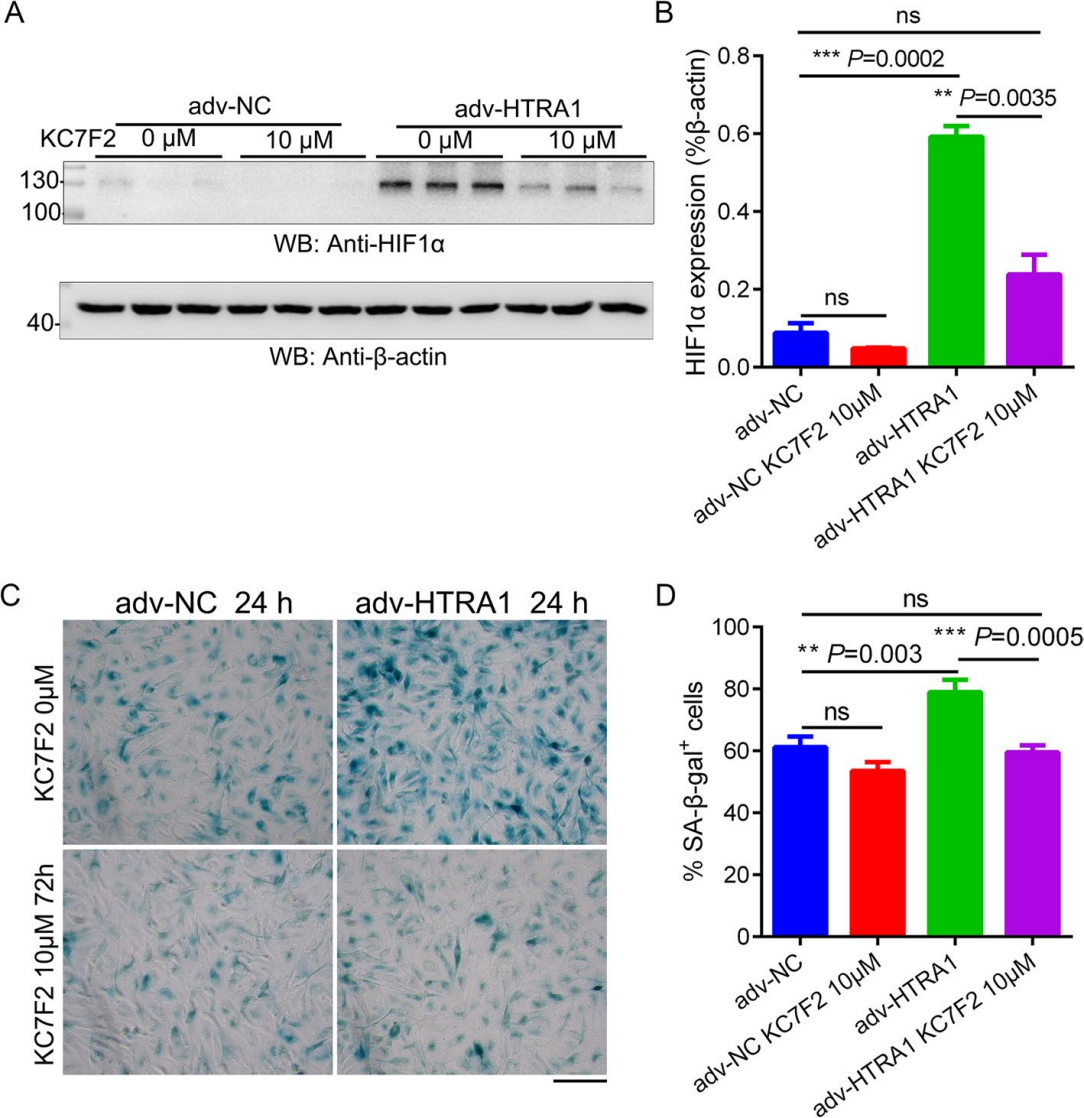
HIF1α translation inhibitor KC7F2 could halt HTRA1-enhanced cellular senescence. **A** Western blot analysis of HIF1α expression in ARPE-19 cells treated with adv-NC or adv-HTRA1 in the presence of HIF1α inhibitor KC7F2. **B** Statistical analysis of the relative expression of HIF1α. **C** SA-β-gal staining of ARPE-19 cells treated with adv-NC or adv-HTRA1 in the presence of HIF1α inhibitor KC7F2 (the scale bar is 50 μm, *n* = 3). **D** Statistical analysis of the positive staining cells in each group of C

The RNA-seq revealed an overlap between HTRA1-induced differentially expressed genes associated with aging and those involved in mitochondrial function and hypoxia response in ARPE-19 cells. To clarify the relationship between HTRA1-induced cellular senescence and cellular hypoxia, we treated the ARPE-19 cells with KC7F2, a HIF1α translation inhibitor, in the presence of HTRA1 overexpression. The upregulation of HIF1α induced by overexpressed HTRA1 can be repressed by KC7F2 (Fig. 7A-B), as well as the cellular senescence (Fig. 7C-D).

### HIF1α translation inhibitor KC7F2 could improve the retinal degeneration induced by NaIO_3_in hHTRA1-Tg mice

**Fig. 8:**
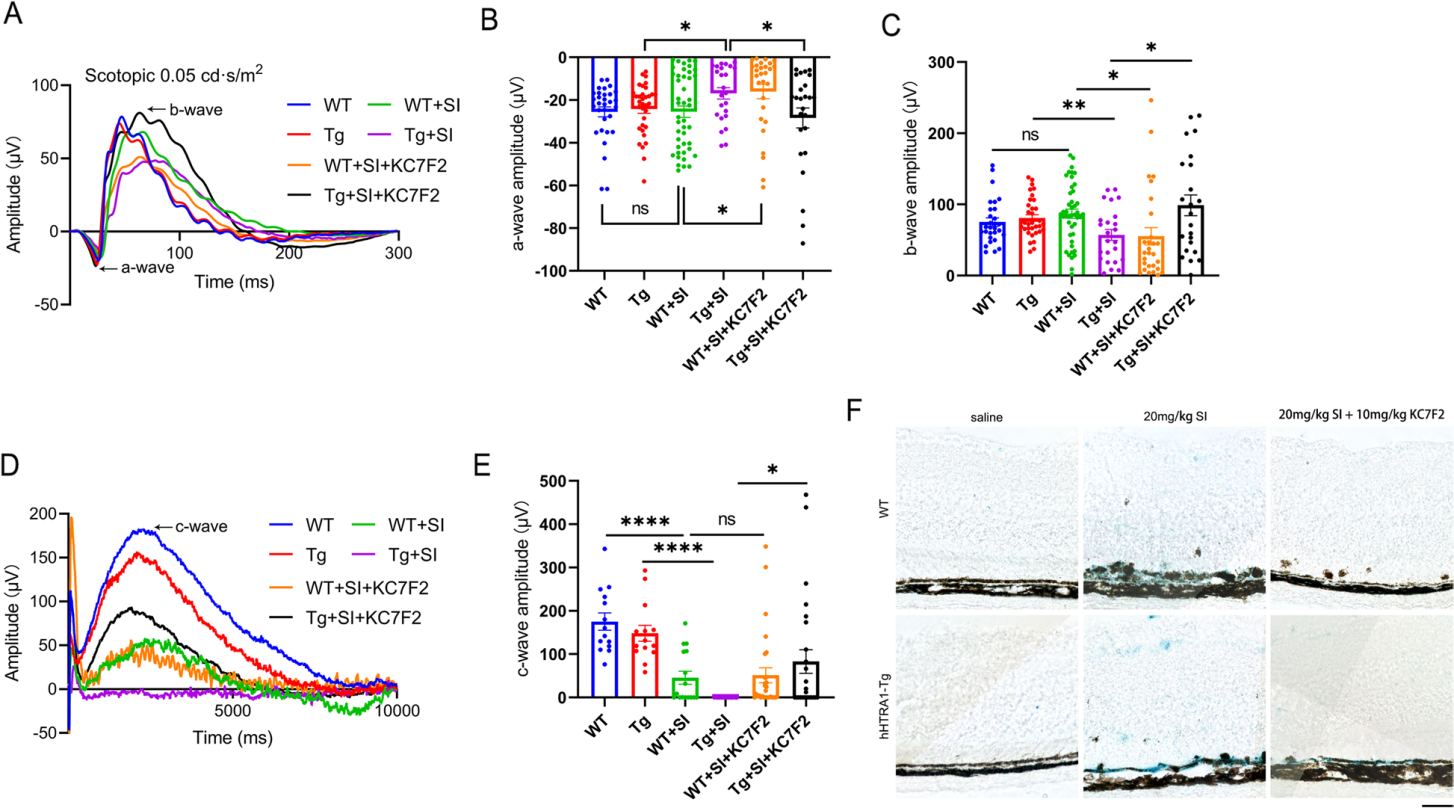
The retinal degeneration induced by NaIO3 could be improved using HIF1α translation inhibitor KC7F2 in hHTRA1-Tg mice. **A** Representative a-wave and b-wave ERG traces of 6- to 10-week-old WT and hHTRA1-Tg mice on the fourth day after intraperitoneal injection of 20mg/kg NaIO3 with or without KC7F2 treatment. WT (*n* = 16), Tg (n = 17), WT+SI (*n* = 22), Tg+SI (*n* = 12), WT+SI+KC7F2 (*n* = 14), Tg+SI+KC7F2 (*n* = 13). Flash intensity was 0.05 cd·s/m2. **B** Statistical analysis of a-wave amplitudes of the 6 groups. **C** Statistical analysis of b-wave amplitudes of the 6 groups. **D** Representative c-wave ERG traces of the 6 groups. WT (*n* = 7), Tg (*n* = 7), WT+SI (*n* = 8), Tg+SI (*n* = 9), WT+SI+KC7F2 (*n* = 14), Tg+SI+KC7F2 (*n* = 13). Flash intensity was 150 cd·s/m2. **E** Statistical analysis of c-wave amplitudes of the 6 groups. **F** Retinal SA-β-gal staining in 6- to 10-week-old WT and hHTRA1-Tg mice on the fourth day after intraperitoneal injection of 20mg/kg NaIO3 with or without KC7F2 treatment (the scale bar is 50μm, *n* = 6)

According to the results in ARPE-19 cells that KC7F2 could reduce the HIF1α expression induced by HTRA1 and halt the cellular senescence, we explored whether KC7F2 could improve retinal degeneration induced by NaIO_3_*in-vivo*. After treated with KC7F2 and 4 days after intraperitoneal injection of 20 mg/kg NaIO_3_, the a-wave, b-wave and c-wave of scotopic flash ERG were improved in hHTRA1-Tg mice compared to hHTRA1-Tg mice only treated with NaIO_3_ (Fig. 8A-E), which indicated that KC7F2 could improve the visual function and alleviate RPE damage in hHTRA1-Tg mice treated with 20 mg/kg NaIO_3_. We’ve also detected retinal senescence in this condition and found KC7F2 could partially inhibit the RPE senescence induced by NaIO_3_ (Fig. 8F).

## Discussion

As a complex disease, AMD is a result of the interplay of aging, environmental factors and genetic factors [25, 26]. *HTRA1* is one of the most significant susceptibility genes in AMD [3]. Oxidative stress, which is increasing with age, is one of the major environmental risk factors for developing AMD [27–29]. In our study, oxidative stress-induced degeneration in mice retina and RPE using sodium iodate (NaIO_3_) was applied to resemble dry AMD [20, 21]. Compared to WT mice, more severe retinal degeneration was observed in hHTRA1-Tg transgenic mice with treatment of NaIO_3_, which showed that the hHTRA1-Tg mice were more susceptible to NaIO_3_ in the development of retinal degeneration.

RPE senescence plays a key role in pathogenesis of AMD [12, 13]. Both cellular senescence and mitochondrial dysfunction are classical hallmarks of the aging process [30]. It has been reported that oxidative stress promoted HTRA1 expression and accelerated ARPE-19 cell senescence [31]. Also, decreased cell senescence has been observed in the HTRA1-deficient cells [32, 33]. In our study, we proved that HTRA1 overexpression caused mitochondrial dysfunction and aggravated cellular senescence of RPE both in vitro and in vivo. Increased p16 and Il-1β in the RPE-choroid of hHTRA1-Tg mice ages 6 to 10 weeks, vacuolated changes in mitochondria as well as enhanced SA-β-gal staining of RPE in hHTRA1-Tg mice ages 12 months were observed. Impaired mitochondrial respiration and promoted glycolysis, increased ROS level and DNA damage, upregulated genes involved in SASP (p21, IL-6 and IL-1β), and SA-β-gal accumulation were detected in ARPE-19 cells with HTRA1 overexpressing. Thus, our study showed that elevated HTRA1 could significantly facilitate RPE senescence.

Growing evidence shows that hypoxia has been implicated in the development and progression of AMD [34–36]. Hypoxia could modulate senescence in a variety of ways, including impairing mitochondrial function, upregulating oxidative stress, causing DNA damage, inducing cell cycle arrest, and triggering inflammatory response [37]. Our study demonstrated that elevated HTRA1 could induce hypoxia in RPE cells. We identified HIF-1 signaling pathway was activated by upregulated HTRA1 in ARPE-19 cells by RNA-seq. We also found that HTRA1 overexpression promoted ARPE-19 cells’ hypoxia, triggered HIF1α expression which mainly located in the nucleus. Thus, HTRA1 was identified as a regulator in promoting cellular hypoxia and HIF-1 signaling.

The elimination of senescent cells is a potential novel therapeutic approach for the treatment of AMD [38–40]. We found cell senescence and impaired mitochondrial respiration induced by elevated HTRA1 can be halted by HIF1α inhibitor KC7F2, suggesting that HTRA1 regulated cell senescence through HIF1α mediated signaling pathway. Importantly, KC7F2 could improve the visual function in hHTRA1-Tg mice treated with NaIO_3_. Therefore, these results indicated that inhibiting HIF-1 signaling might be a potential treatment for AMD.

There are some limitations in our study. Both hHTRA1 and mHTRA1 were expressed in retina and RPE in hHTRA1-Tg mice. As RPE and photoreceptor are affected cells in mice treated with NaIO_3_, it would be more appropriated to use RPE-specific or rod-specific HTRA1-Tg mouse to detect the primary site affected in our future study. Also, HTRA1 induced RPE senescence under oxidative stress. Age is one of the most important factors in AMD, though oxidative stress was increased with aging, more studies were required to explore the direct correlation between HTRA1 and age-related senescence in elderly mice model.

Taken together, our findings pointed out that HTRA1 plays an essential role in RPE senescence. Upregulation of HTRA1 could significantly enhance hypoxia, then impair mitochondrial respiration and promote ROS production, and subsequently activate HIF-1 signaling, which lead to accelerated RPE senescence. Elevated HTRA1 has been found not only in AMD, but also in age-related frailty and Alzheimer’s disease, two age-related diseases [41, 42]. Our findings will provide new insights for developing potential therapeutic strategies for AMD and other age-related diseases.

## Conclusions

Our study demonstrated that HTRA1 could activate HIF-1 signaling and promote RPE senescence in retinal degeneration. Inhibition of HIF-1 signaling could partially prevent RPE senescence and restore visual function in hHTRA1-Tg mice treated with NaIO_3_. Our study will lead to a better understanding of AMD pathogenesis and provide potential treatment for AMD.

## Supplementary Information

**Additional file 1: Figure S1.** Genotyping of transgenic mice with human HTRA1 knock in. **Table S1.** The primers for genotyping of WT mice and transgenic mice with human HTRA1 knock in. **Figure S2.** HTRA1 overexpression in hHTRA1-Tg mice. **Figure S3.** Little obvious retinal degeneration was observed in hHTRA1-Tg mice. **Figure S4.** Retinal histologyof 8-week and 12-month-old WT and hHTRA1-Tg mice. **Table S2.** The primers for RT-qPCR.  **Figure S5.** The phenotype of hHTRA1-Tg and WT mice were the same when treated with 10 mg/kg and 35 mg/kg NaIO_3_. **Figure S6.** HTRA1 in retina did not response to the SI induced stress. **Figure S7.** HTRA1 overexpression aggravated DNA damage of primary mouse RPE cells. **Figure S8.** The mitochondrial ROS production did not increase significantly after 24 h treatment of adv-HTRA1. **Figure S9.** HTRA1 induced HIF1α expression in primary mouse RPE cells. 

## Data Availability

The data that support the findings of this study are available from the corresponding author. The RNA-seq data were deposited in gene expression omnibus (GEO), Accession No. GSE 186816.

## Abbreviations

AMD: Age-related macular degeneration
RPE: Retinal pigment epithelium
GWAS: Genome-wide association studies
HTRA1: High-temperature requirement A1
HIF: Hypoxia-inducible factor
CNV: Choroidal neovascularization
ERG: Electroretinogram
FFA: Fundus photography, fundus fluorescein angiography
SD-OCT: Spectral-domain optical coherence tomography
TEM: Transmission electron microscopy
OCR: Oxygen consumption rate
ECAR: Extracellular acidification rate
SA-β-gal: Senescence-associated-β-galactosidase
ROS: Reactive oxygen species
SASP: Senescence-associated secretory phenotype

## References

[1] Mitchell P (2018). Age-related macular degeneration. Lancet.

[2] Apte RS (2021). Age-related Macular Degeneration. N Engl J Med.

[3] Fritsche LG (2016). A large genome-wide association study of age-related macular degeneration highlights contributions of rare and common variants. Nat Genet.

[4] Eigenbrot C (2012). Structural and functional analysis of HtrA1 and its subdomains. Structure.

[5] Grassmann F (2017). Recombinant haplotypes narrow the ARMS2/HTRA1 Association Signal for Age-Related Macular Degeneration. Genetics.

[6] Yang Z (2006). A variant of the HTRA1 gene increases susceptibility to age-related macular degeneration. Science.

[7] Chan CC, et al. *Human HtrA1 in the archived eyes with age-related macular degeneration*. Trans Am Ophthalmol Soc, 2007. 105: p. 92 – 7; discussion 97 – 8. PMC2258134 18427598

[8] Tosi GM (2017). HTRA1 and TGF-beta1 concentrations in the aqueous humor of patients with Neovascular Age-Related Macular Degeneration. Invest Ophthalmol Vis Sci.

[9] Jones A (2011). Increased expression of multifunctional serine protease, HTRA1, in retinal pigment epithelium induces polypoidal choroidal vasculopathy in mice. Proc Natl Acad Sci U S A.

[10] Khanani AM (2021). Phase 1 study of the Anti-HtrA1 antibody-binding Fragment FHTR2163 in Geographic Atrophy secondary to age-related Macular Degeneration. Am J Ophthalmol.

[11] Tom I (2020). Development of a therapeutic anti-HtrA1 antibody and the identification of DKK3 as a pharmacodynamic biomarker in geographic atrophy. Proc Natl Acad Sci U S A.

[12] Kozlowski MR (2012). RPE cell senescence: a key contributor to age-related macular degeneration. Med Hypotheses.

[13] Lee KS (2021). Cellular senescence in the aging retina and developments of senotherapies for age-related macular degeneration. J Neuroinflammation.

[14] Ahamed W, et al. HTRA1 regulates subclinical inflammation and activates proangiogenic response in the retina and choroid. Int J Mol Sci. 2022;23(18). 10.3390/ijms231810206. 10.3390/ijms231810206 PMC9499640 36142120

[15] Visuvanathan S (2022). XIAP gene therapy effects on retinal ganglion cell structure and function in a mouse model of glaucoma. Gene Ther.

[16] Chen M (2013). Age- and light-dependent development of localised retinal atrophy in CCL2(-/-)CX3CR1(GFP/GFP) mice. PLoS ONE.

[17] Dunn KC, et al. ARPE-19, a human retinal pigment epithelial cell line with differentiated properties. Exp Eye Res. 1996;62(2). 10.1006/exer.1996.0020. 155 – 69. 10.1006/exer.1996.0020 8698076

[18] Fernandez-Godino R, Garland DL, Pierce EA (2016). Isolation, culture and characterization of primary mouse RPE cells. Nat Protoc.

[19] Zhao M (2021). Defect of LSS disrupts Lens Development in Cataractogenesis. Front Cell Dev Biol.

[20] Wang J (2014). Direct effect of sodium iodate on neurosensory retina. Invest Ophthalmol Vis Sci.

[21] Koster C, et al. Sodium-Iodate Injection Can replicate retinal degenerative Disease Stages in pigmented mice and Rats: non-invasive Follow-Up using OCT and ERG. Int J Mol Sci. 2022;23(6). 10.3390/ijms23062918. 10.3390/ijms23062918 PMC8953416 35328338

[22] Moiseeva O, et al. Mitochondrial dysfunction contributes to oncogene-induced senescence. Mol Cell Biol. 2009;29(16). 10.1128/MCB.01868-08. 4495 – 507. 10.1128/MCB.01868-08 PMC2725737 19528227

[23] Wiley CD (2016). Mitochondrial dysfunction induces senescence with a distinct secretory phenotype. Cell Metab.

[24] Fearon U (2016). Hypoxia, mitochondrial dysfunction and synovial invasiveness in rheumatoid arthritis. Nat Rev Rheumatol.

[25] Kuan V (2021). Association of Smoking, Alcohol Consumption, blood pressure, body Mass Index, and glycemic risk factors with age-related Macular Degeneration: a mendelian randomization study. JAMA Ophthalmol.

[26] Wang W (2016). Genetic and environmental factors strongly influence risk, severity and progression of age-related macular degeneration. Signal Transduct Target Ther.

[27] Abokyi S, et al. *Central Role of Oxidative Stress in Age-Related Macular Degeneration: Evidence from a Review of the Molecular Mechanisms and Animal Models* Oxid Med Cell Longev, 2020. 2020: p. 7901270. 10.1155/2020/7901270. 10.1155/2020/7901270 PMC7035553 32104539

[28] Datta S (2017). The impact of oxidative stress and inflammation on RPE degeneration in non-neovascular AMD. Prog Retin Eye Res.

[29] Liguori I (2018). Oxidative stress, aging, and diseases. Clin Interv Aging.

[30] Chapman J, Fielder E, Passos JF (2019). Mitochondrial dysfunction and cell senescence: deciphering a complex relationship. FEBS Lett.

[31] Supanji (2013). HtrA1 is induced by oxidative stress and enhances cell senescence through p38 MAPK pathway. Exp Eye Res.

[32] Schillinger J (2018). HTRA1-Dependent cell cycle proteomics. J Proteome Res.

[33] Schmidt N (2016). Epigenetic silencing of serine protease HTRA1 drives polyploidy. BMC Cancer.

[34] Blasiak J (2014). Oxidative stress, hypoxia, and autophagy in the neovascular processes of age-related macular degeneration. Biomed Res Int.

[35] Kurihara T, et al. Hypoxia-induced metabolic stress in retinal pigment epithelial cells is sufficient to induce photoreceptor degeneration. Elife. 2016;5. 10.7554/eLife.14319. 10.7554/eLife.14319 PMC4848091 26978795

[36] Stefansson E, Geirsdottir A, Sigurdsson H (2011). Metabolic physiology in age related macular degeneration. Prog Retin Eye Res.

[37] Welford SM, Giaccia AJ (2011). Hypoxia and senescence: the impact of oxygenation on tumor suppression. Mol Cancer Res.

[38] Chae JB (2021). Targeting senescent retinal pigment epithelial cells facilitates retinal regeneration in mouse models of age-related macular degeneration. Geroscience.

[39] Naylor RM, Baker DJ, van Deursen JM (2013). Senescent cells: a novel therapeutic target for aging and age-related diseases. Clin Pharmacol Ther.

[40] Zhang L, et al. Cellular senescence: a key therapeutic target in aging and diseases. J Clin Invest. 2022;132(15). 10.1172/JCI158450. 10.1172/JCI158450 PMC9337830 35912854

[41] Grau S (2005). Implications of the serine protease HtrA1 in amyloid precursor protein processing. Proc Natl Acad Sci U S A.

[42] Lorenzi M (2016). Association of frailty with the serine protease HtrA1 in older adults. Exp Gerontol.

