# Supplementary material for: Inhibiting HIF-1 signaling alleviates HTRA1-induced RPE senescence in retinal degeneration
Source: Cell Commun Signal. 2023 Jun 14;21:134. doi: 10.1186/s12964-023-01138-9 (PMC10265780; doi:10.1186/s12964-023-01138-9)
Supplement: Supplementary file 2 — Additional file 1: Figure S1. Genotyping of transgenic mice with human HTRA1 knock in. Table S1. The primers for genotyping of WT mice and transgenic mice with human HTRA1 knock in. Figure S2. HTRA1 overexpression in hHTRA1-Tg mice. Figure S3. Little obvious retinal degeneration was observed in hHTRA1-Tg mice. Figure S4. Retinal histologyof 8-week and 12-month-old WT and hHTRA1-Tg mice. Table S2. The primers for RT-qPCR. Figure S5. The phenotype of hHTRA1-Tg and WT mice were the same when treated with 10 mg/kg and 35 mg/kg NaIO3. Figure S6. HTRA1 in retina did not response to the SI induced stress. Figure S7. HTRA1 overexpression aggravated DNA damage of primary mouse RPE cells. Figure S8. The mitochondrial ROS production did not increase significantly after 24 h treatment of adv-HTRA1. Figure S9. HTRA1 induced HIF1α expression in primary mouse RPE cells. [file 12964_2023_1138_MOESM1_ESM.docx]

**
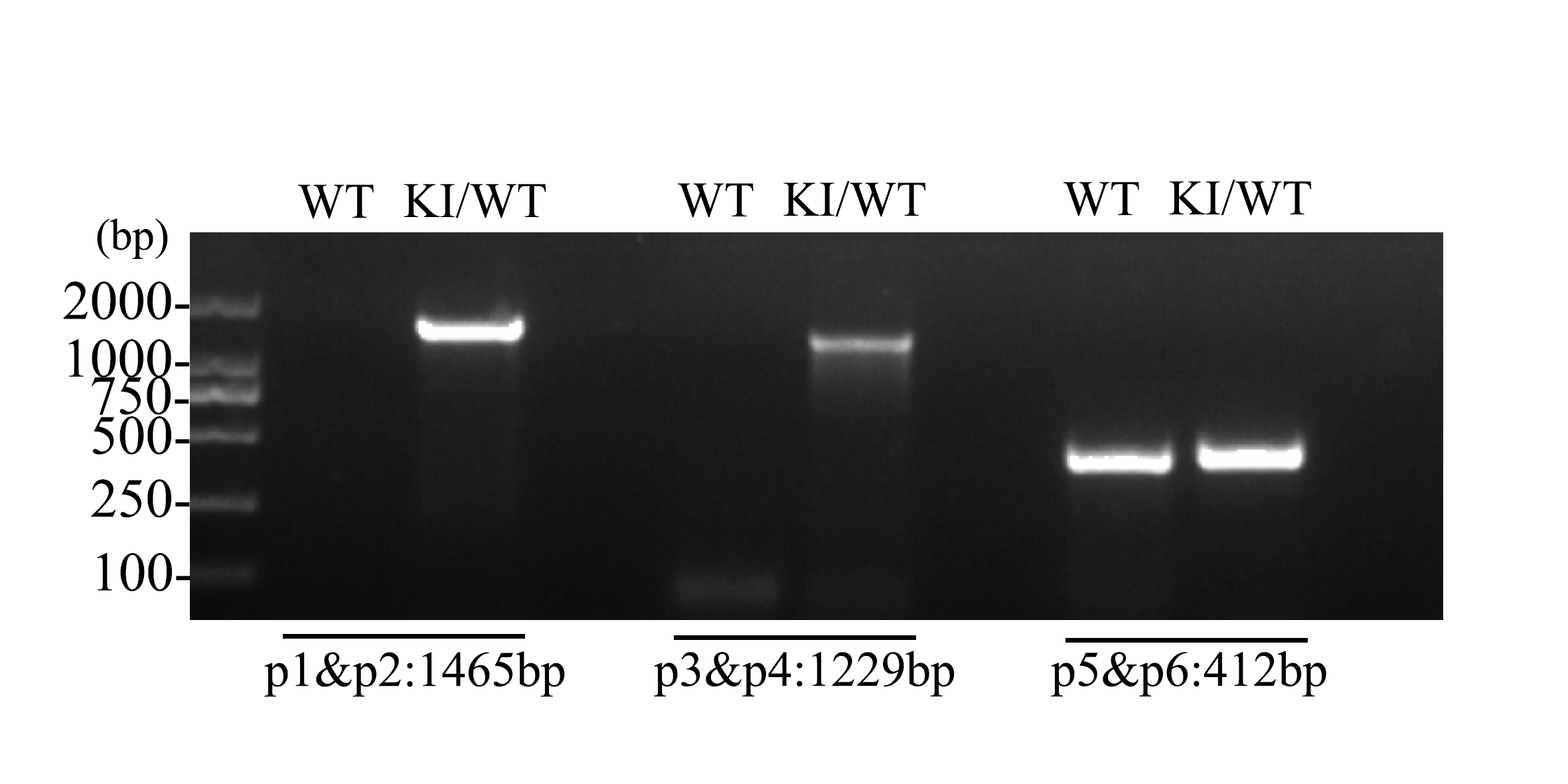
**

**Figure S1. Genotyping of transgenic mice with human *HTRA1* knock in (KI).** Founders and transgene-positive hHTRA1-Tg mice were identified by PCR of genomic DNA obtained from tail biopsies with primers in Table S1.

**Table S1. The primers for genotyping of WT mice and transgenic mice with human HTRA1 knock in.**

|  | **Primer name** | **Sequence(5’-3’)** | **Size (bp)** |
| --- | --- | --- | --- |
| Primer pair 1 | p1 | ATGCCCACCAAAGTCATCAGTGTAG | WT: none |
|  | p2 | AGGCGGGCCATTTACCGTAAGTTA | KI: 1456 |
| Primer pair 2 | p3 | CCTCCTCTCCTGACTACTCCCAGTC | WT: none |
|  | p4 | TCACAGAAACCATATGGCGCTCC | KI: 1229 |
| Primer pair 3 | p5 | CAGCAAAACCTGGCTGTGGATC | WT: 412 |
|  | p6 | ATGAGCCACCATGTGGGTGTC | KI: 412 |


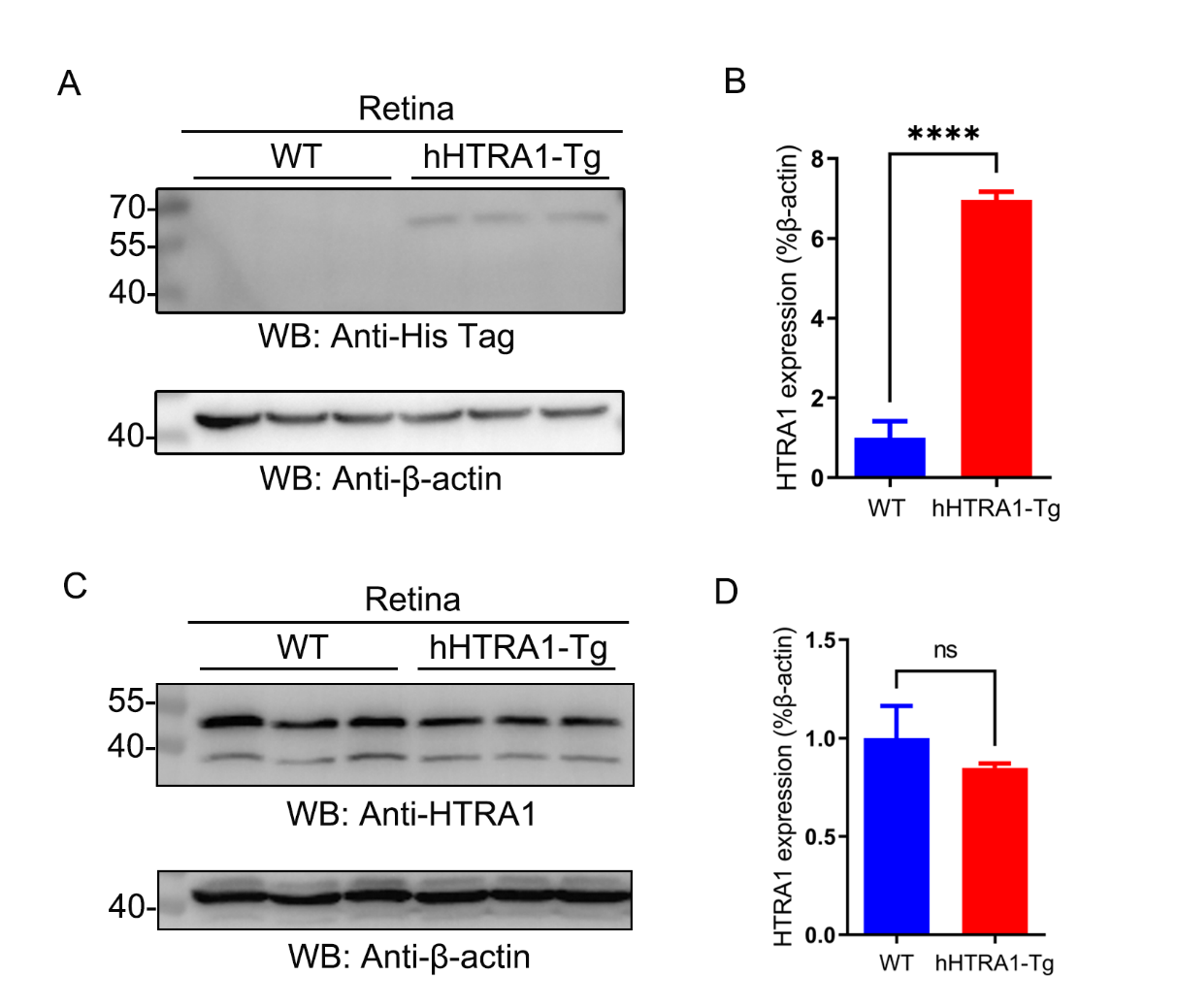


**Figure S2. HTRA1 overexpression in hHTRA1-Tg mice.** A. Western blot analysis of hHTRA1 expression in retina of 6- to 8-week-old WT and hHTRA1-Tg mice using anti-His antibody. B. Statistical analysis of the relative expression of hHTRA1 in retina (*P*<0.0001). C. Western blot analysis of mHTRA1 expression in retina of 6- to 8-week-old WT and hHTRA1-Tg mice using HTRA1 antibody. D. Statistical analysis of the relative expression of mHTRA1 in retina (*P*=0.187).


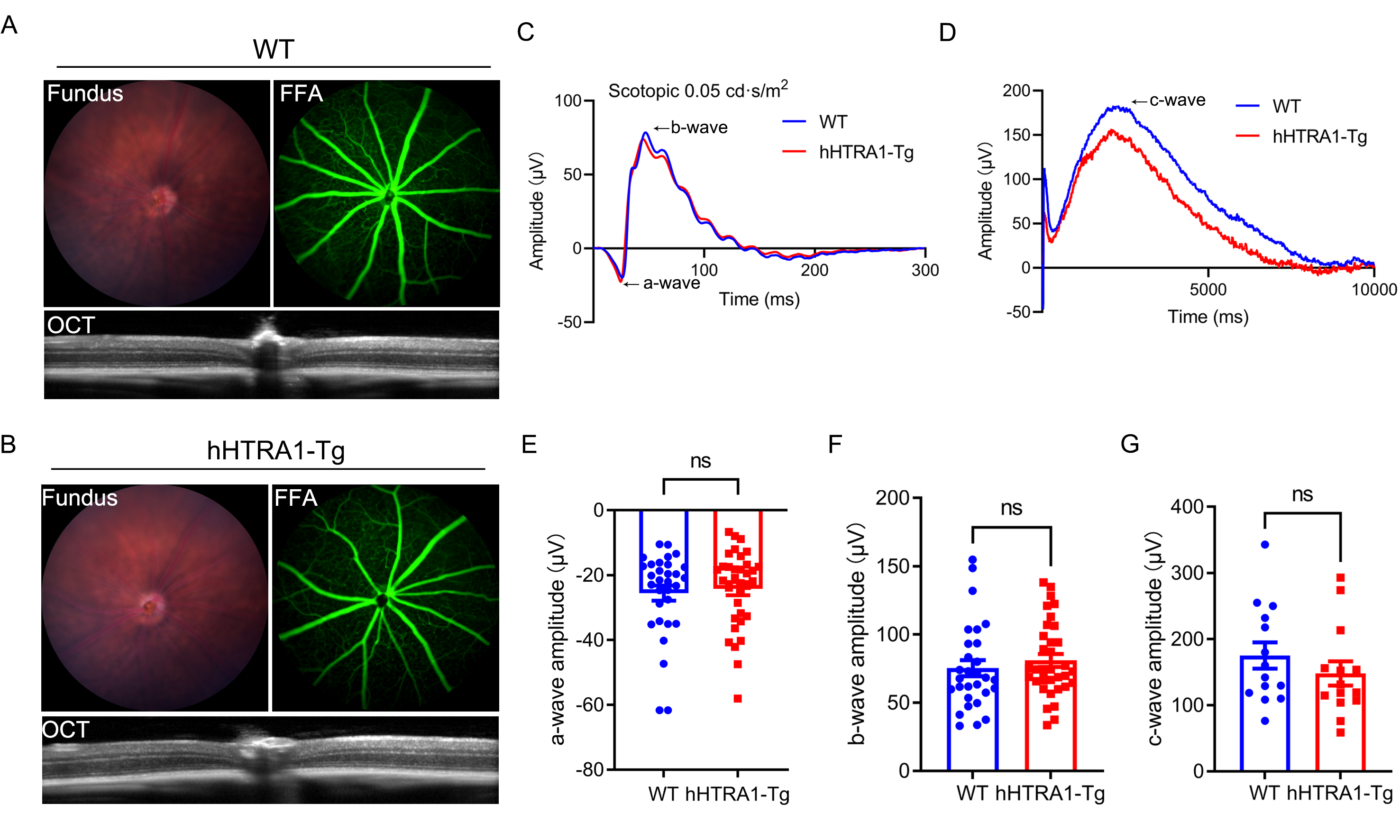


**Figure S3. Little obvious retinal degeneration was observed in hHTRA1-Tg mice.** A. Representative fundus image, FFA and OCT of WT mice. B. Representative fundus image, FFA and OCT of hHTRA1-Tg mice. C. Representative a-wave and b-wave ERG traces of 6- to 10-week-old hHTRA1-Tg (n = 17) and WT mice (n = 16) . Flash intensity was 0.05 cd·s/m^2^. D. Representative c-wave traces of 6- to 10-week-old hHTRA1-Tg (n = 7) and WT mice (n = 7). Flash intensity was 150 cd·s/m^2^. E. Statistical analysis of a-wave amplitudes for WT and hHTRA1-Tg mice (*P*=0.657). F. Statistical analysis of b-wave amplitudes for WT and hHTRA1-Tg mice (*P*=0.447). G. Statistical analysis of c-wave amplitudes for WT and hHTRA1-Tg mice (*P*=0.326).

**
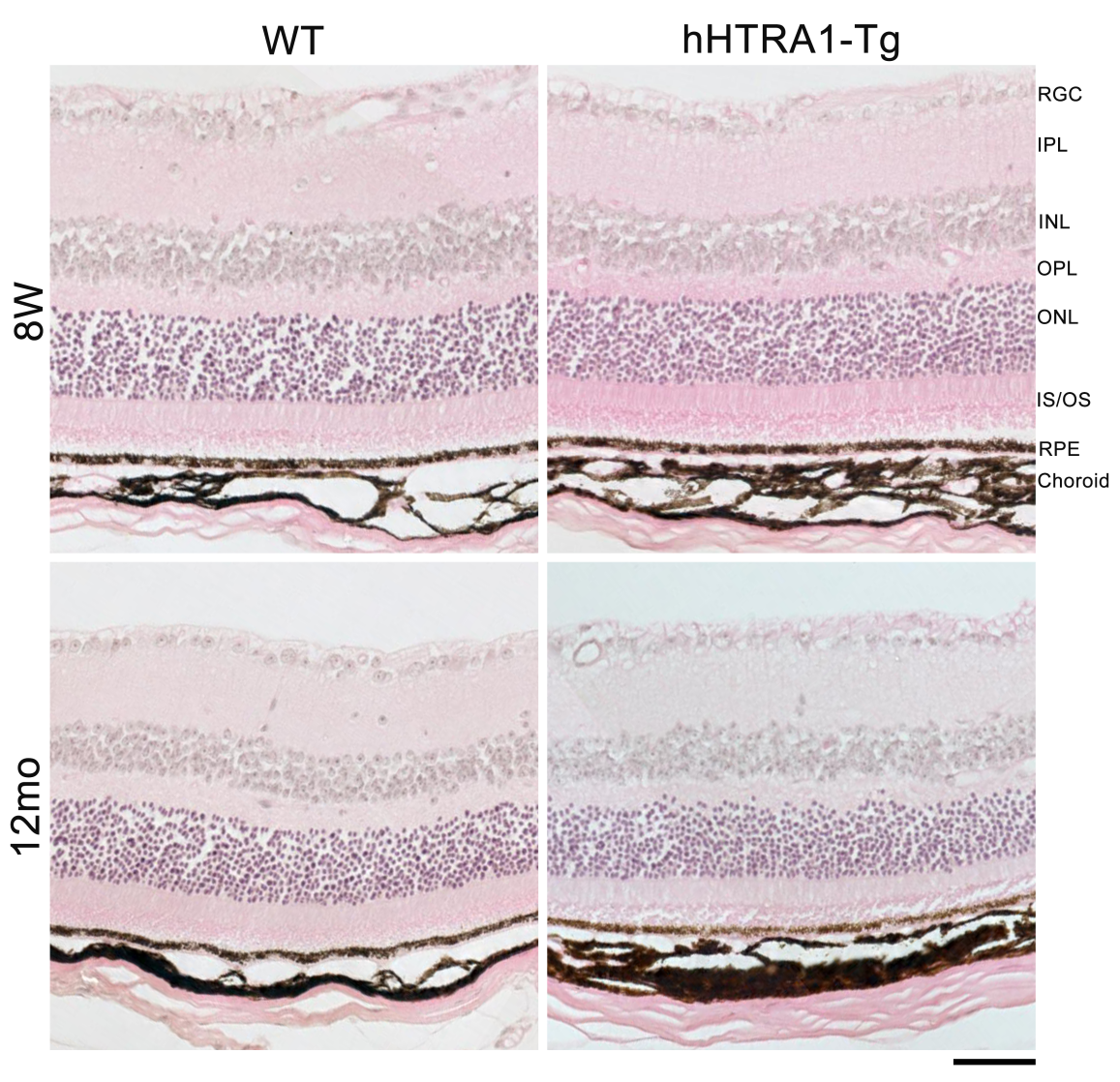
**

**Figure S4. Retinal histology (H&E staining) of 8-week and 12-month-old WT and hHTRA1-Tg mice.** The scale bar is 50μm（n = 3）. RGC, retinal ganglion cell; IPL, inner plexiform layer; INL, inner nuclear layer; OPL, outer plexiform layer; ONL, outer nuclear layer; IS, photoreceptor inner segment; OS, photoreceptor outer segment; RPE, retinal pigment epithelium.

**Table S2. The primers for RT-qPCR.**

|  | **Primer name** | **Sequence (5’-3’)** |
| --- | --- | --- |
|  | p21-Forward | AGGTGGACCTGGAGACTCTCAG |
|  | p21- Reverse | TCCTCTTGGAGAAGATCAGCCG |
|  | IL-6- Forward | AGACAGCCACTCACCTCTTCAG |
|  | IL-6- Reverse | TTCTGCCAGTGCCTCTTTGCTG |
|  | IL-1β- Forward | CCACAGACCTTCCAGGAGAATG |
| human | IL-1β- Reverse | GTGCAGTTCAGTGATCGTACAGG |
|  | SOD2- Forward | CTGGACAAACCTCAGCCCTAAC |
|  | SOD2- Reverse | AACCTGAGCCTTGGACACCAAC |
|  | MMP2- Forward | AGCGAGTGGATGCCGCCTTTAA |
|  | MMP2- Reverse | CATTCCAGGCATCTGCGATGAG |
|  | SESN2- Forward | AGATGGAGAGCCGCTTTGAGCT |
|  | SESN2- Reverse | CCGAGTGAAGTCCTCATATCCG |
|  | GAPDH-Forward | TCACCATCTTCCAggAgCgA |
|  | GAPDH-Reverse | CTTCTCCATggTggTgAAgAC |
|  | p16- Forward | TGTTGAGGCTAGAGAGGATCTTG |
| mouse | p16- Reverse | CGAATCTGCACCGTAGTTGAGC |
|  | IL-1b- Forward | TGGACCTTCCAGGATGAGGACA |
|  | IL-1b- Reverse | GTTCATCTCGGAGCCTGTAGTG |
|  | GAPDH-Forward | GTGAAGGTCGGTGTGAACGG |
|  | GAPDH-Reverse | GCCGTTGAATTTGCCGTGAG |


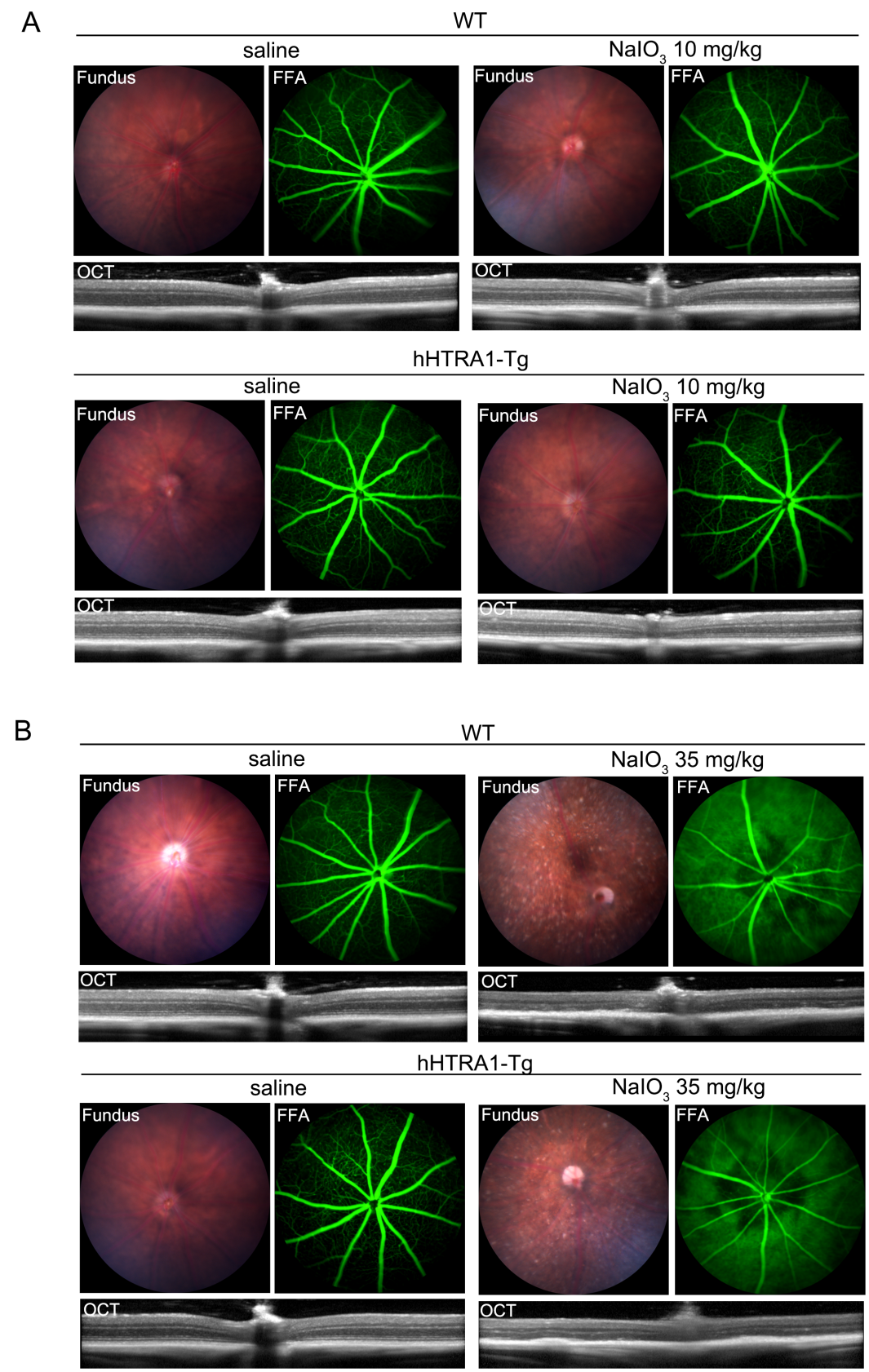


**Figure S5. The phenotype of hHTRA1-Tg and WT mice were the same when treated with 10 mg/kg and 35 mg/kg NaIO_3_.** A. Representative fundus image, FFA and OCT of WT and hHTRA1-Tg mice with injection of 10 mg/kg NaIO_3_ and similar volumes of physiological saline (n=10). B. Representative fundus image, FFA and OCT of WT and hHTRA1-Tg mice with injection of 35 mg/kg NaIO_3_ and similar volumes of physiological saline (n=10).


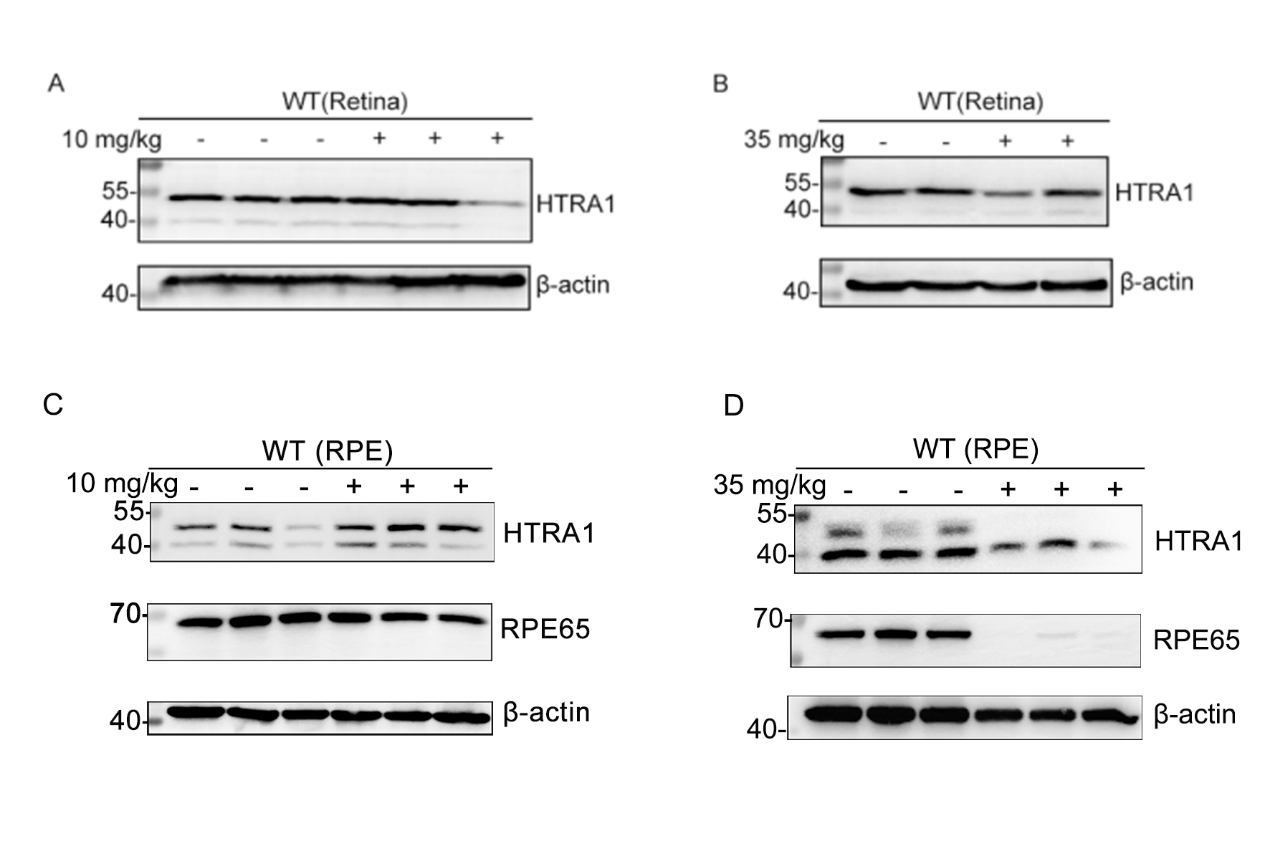


**Figure S6. HTRA1 in retina did not response to the SI induced stress.** A. Western blot analysis of HTRA1 expression in retina of WT mice treated with 10 mg/kg SI or saline. B. Western blot analysis of HTRA1 expression in retina of WT mice treated with 35 mg/kg SI or saline. C. Western blot analysis of HTRA1 and RPE65 expression in RPE-choroid of WT mice treated with 10 mg/kg SI or saline. D. Western blot analysis of HTRA1 and RPE65 expression in RPE-choroid of WT mice treated with 35 mg/kg SI or saline.


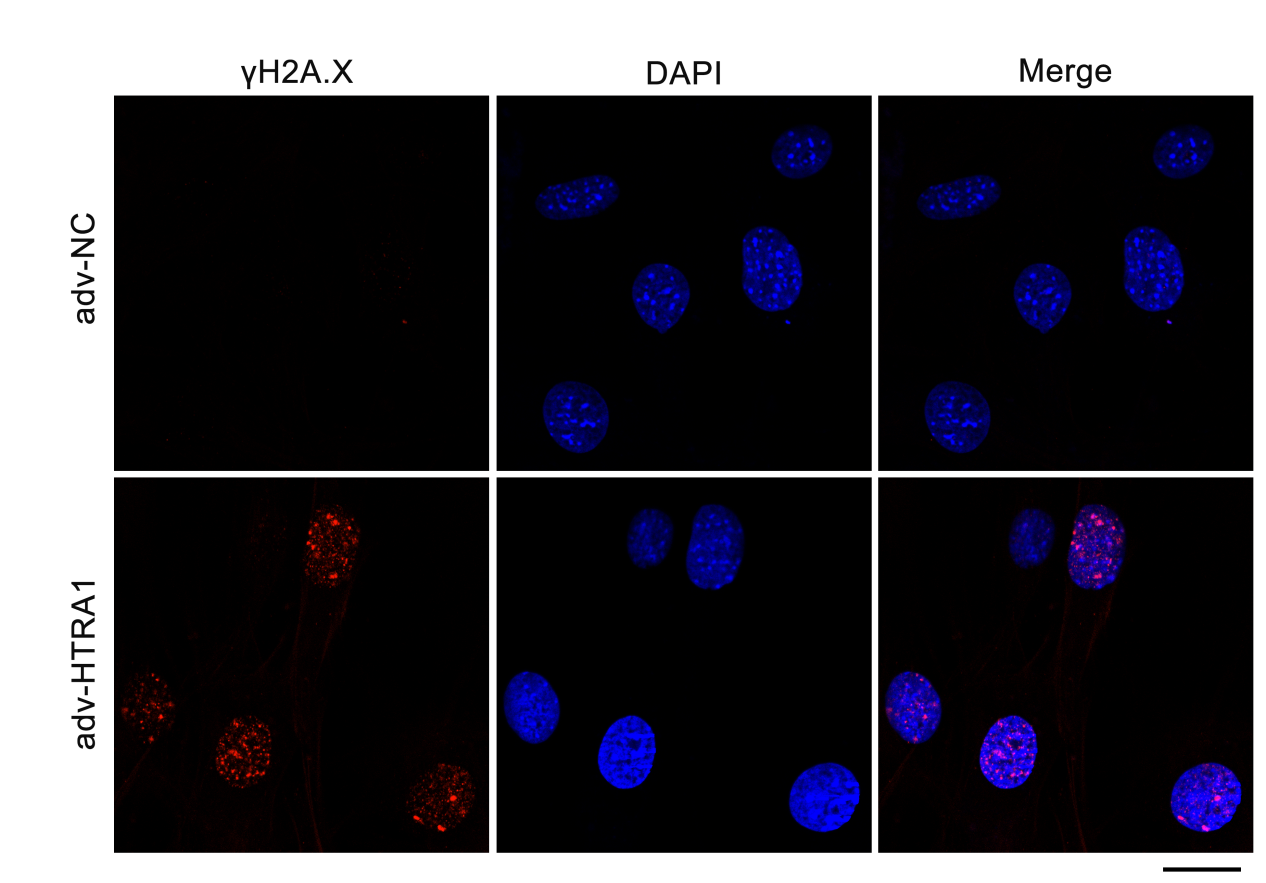


**Figure S7. HTRA1 overexpression aggravated DNA damage of primary mouse RPE cells.** Immunofluorescent analysis of DNA damage by detecting the expression of γH2A.X in primary mouse RPE cells treated with adv-NC or adv-HTRA1 for 48 h (the scale bar is 20 μm, n = 3).


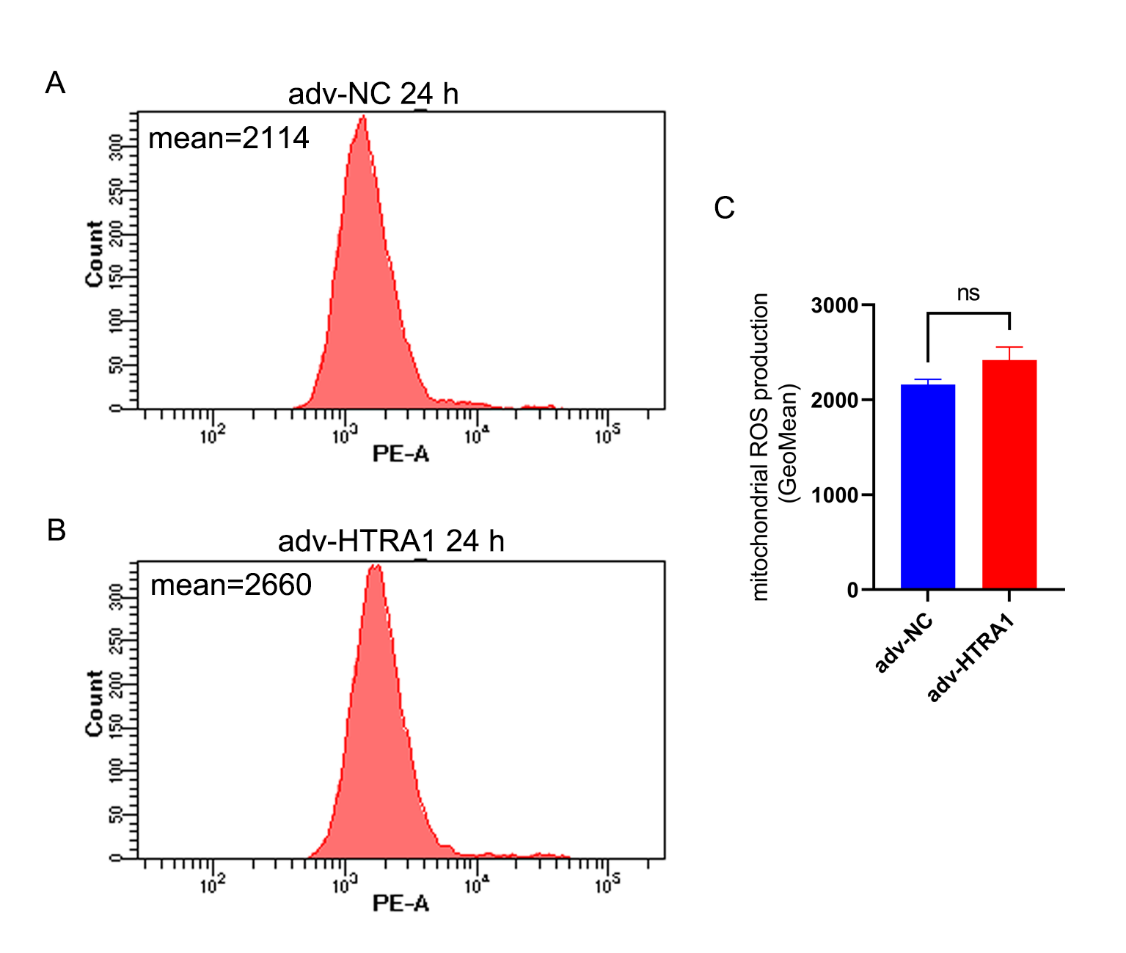


**Figure S8. The mitochondrial ROS production did not increase significantly** **after 24 h treatment of adv-****HTRA1.** A. Flow cytometry detection of ROS in ARPE-19 cells treated with adv-NC for 24 h (n = 3). B. Flow cytometry detection of ROS in ARPE-19 cells treated with adv-HTRA1 for 24 h (n = 3). C. Statistical analysis of the mitochondrial ROS production.


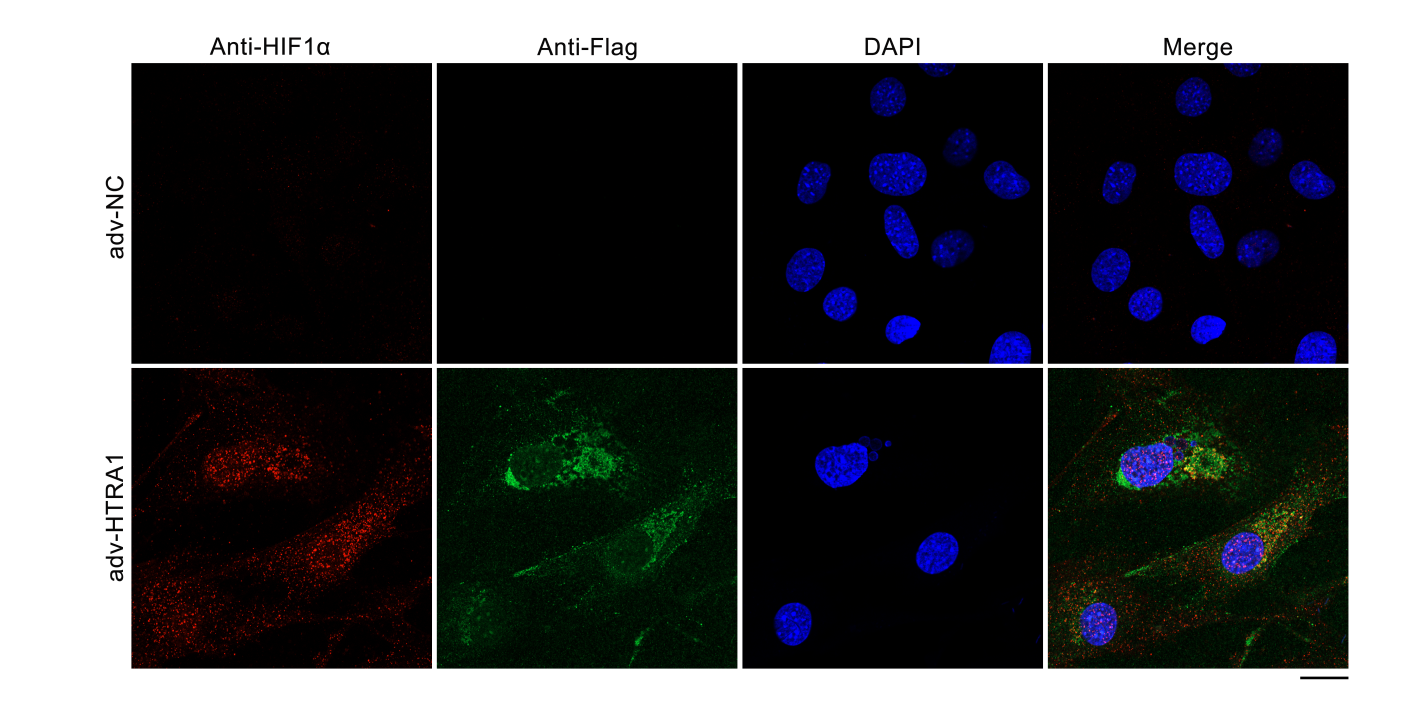


**Figure S9. HTRA1 induced HIF1α expression in primary mouse RPE cells.** Immunofluorescent analysis of HIF1α expression in primary mouse RPE cells treated with adv-NC or adv-HTRA1 for 48 h (the scale bar is 20 μm, n = 3). Adv-HTRA1 was fused with flag.
